# Classification of *Isatis indigotica* Fortune and *Isatis tinctoria* Linnaeus via comparative analysis of chloroplast genomes

**DOI:** 10.1186/s12864-023-09534-8

**Published:** 2023-08-18

**Authors:** Yong Su, Man Zhang, Qiaosheng Guo, Min Wei, Hongzhuan Shi, Tao Wang, Zhengzhou Han, Huihui Liu, Chang Liu, Jianmin Huang

**Affiliations:** 1 Institute of Chinese Medicinal Materials, Nanjing Agricultural University, Nanjing City, 210095 Jiangsu Province PR China; 2 China Resources Sanjiu Medical & Pharmaceutical Co., Ltd, Shenzhen City, 518000 PR China

**Keywords:** Chloroplast genome, Germplasm, *Isatis indigotica* Fortune, Autotetraploid

## Abstract

**Background:**

*Isatis tinctoria* Linnaeus and *Isatis indigotica* Fortune are very inconsistent in their morphological characteristics, but the *Flora of China* treats them as the same species. In this work, a new technology that differs from conventional barcodes is developed to prove that they are different species and to clarify their classification.

**Results and methods:**

*I. indigotica* was indistinguishable from *I. tinctoria* when using ITS2. CPGAVAS2 was used to construct the chloroplast genomes. MAFFT and DnaSP were used to calculate nucleotide polymorphism, the chloroplast genomes of the two have high diversity in the *rpl*32 ~ *trn*L-UAG short region. When using this region as a mini barcode, it was found that there are obvious differences in the base numbers of *I. tinctoria* and different ploidy *I. indigotica* were found, but diploid and tetraploid *I. indigotica* had the same number of bases. Moreover, the reconstruction of the maximum likelihood (ML) tree, utilizing the mini-barcode, demonstrated that *I. tinctoria* and both diploid and tetraploid *I. indigotica* are located on distinct branches. The genome size of tetraploid *I. indigotica* was approximately 643.773 MB, the heterozygosity rate was approximately 0.98%, and the repeat sequence content was approximately 90.43%. This species has a highly heterozygous, extremely repetitive genome.

**Conclusion:**

A new method was established to differentiate between *I. indigotica* and *I. tinctoria*. Furthermore, this approach provides a reference and basis for the directional breeding of *Isatis*.

**Supplementary Information:**

The online version contains supplementary material available at 10.1186/s12864-023-09534-8.

## Introduction

*Isatis indigotica* Fortune is a biennial herb belonging to Brassicaceae [1]. Its dry leaves and roots are Isatidis Folium and Isatidis Radix, respectively [2]. As a traditional Chinese medicine from Shen Nong’s *Materia Medica* used for over 2000 years, it functions in cooling the blood and pharynx, clearing away heat and detoxifying. In addition, the herb treats fever and sore throat, spots due to dampness poisoning, coma, and polydipsia [3, 4]. However, in 2001, the *Flora of China* (*FOC*) merged. *I. indigotica* and the *Isatis tinctoria* Linnaeus into the latter [5] due to their morphological diversity and interspecific transitions; therefore, they should not be considered as two separate species. However, Wang from China investigated the scientific name of *I. indigotica* in 1982 and found that *I. indigotica* and *I. tinctoria* differed greatly in the morphology of seedlings, petals, fruits, rhizomes, etc., theoretically, these may be two different species [6]. Given that accurate species identification of medicinal herbs is an important prerequisite for ensuring the safety and effectiveness of medicinal materials, it is important to understand whether the original plant of Isatidis Radix and Isatidis Folium is *I. indigotica* or *I. tinctoria* or if they are the same species. Further identification is thus needed.

The methods of species identification include morphological identification, genetic material identification and molecular markers. Despite the advantages of simple and rapid morphological identification, the plant phenotype is susceptible to variation resulting from its genetic material and external environment, due to its high level of plasticity [7]; therefore, it is difficult to accurately identify species by morphological methods alone. Since genetic material controls the occurrence of biological forms, detection of the number of nuclei, genome size, DNA content, and chromosome number of organs and tissues by methods such as chromosome counting and flow cytometry has gradually become a reliable approach for plant classification [8]. The disadvantage is that two similar species of the same ploidy cannot be differentiated. Due to their advantages of being unaffected by the environment,highly stable, highly polymorphic, and highly accurate, DNA molecular markers have become the most popular method of identifying plant taxa [1]. However, DNA molecular markers can be analysed only from the perspective of nucleic acids or proteins [8], and cannot directly locate genes or genotypes; therefore, they cannot distinguish two species with extremely similar genomes. However, there have been few reports on diploid and tetraploid genomes. Polyploidy research mainly focuses on transcription and metabolism, and most studies are performed using RNAseq-based transcriptome analysis [9, 10]. In view of the above, there is an urgent need to develop a rapid species identification method to identify two species with extremely similar genotypes.

In this work, we collected 26 samples of diploid *I. indigotica*, 1 sample of tetraploid *I. indigotica*, and 1 sample of *I. tinctoria* in China. The complete chloroplast genome was reconstructed using whole-genome Illumina sequencing data available from the NCBI. Comparing the performance of ITS2 and mini-barcoding for *I. indigotica* and *I. tinctoria*, genome sequencing analysis and evaluation of tetraploid *I. indigotica* can further reveal whether it has a highly heterozygous and highly repetitive genome. Therefore, this study will provide a reference for clarifying the germplasm source of *I. indigotica* and the improving active components, as well as provide a new method for the distinguishing of highly similar species.

## Materials and methods

### Plant material

Diploid seeds of twenty–six *I. indigotica* germplasms (1–26), were collected from Xinjiang, Henan, Anhui, Hebei, Shanxi, and Gansu Provinces in China in 2015 and identified by Dr. Qiaosheng Guo of Nanjing Agricultural University. Tetraploid seeds of *I. indigotica* (27) were provided by Resources Sanjiu Medical & Pharmaceutical Co., Ltd, of China. The *I. tinctoria* seeds (28) were identified by BaharGuli Huangerhan, a researcher with the Food and Drug Inspection Institute of Altay Prefecture in 2015 (Table S1).

All seeds were planted at Nanjing Agricultural University (N = 10). The plant height, leaf length, leaf width, petiole length, number of blades, and single leaf area were measured at the growth and flourishing stages, and the taproot length, taproot width, branch number of the taproot, phloem, xylem and hundred-grain weight were measured at the harvest stage. Silique data including hundred-grain weight, silique length, silique width and the length to width ratio of siliques were collected. The leaf area, xylem and phloem were measured using ImageJ software (V1.8.0.345), and other data were measured by a digital micrometer (Dongguan Sanliang Instrument Co., Ltd., China). All data were analysed by principal component analysis (PCA) performed on the Metware Cloud (https://cloud.metware.cn/#/home). The cor program in RStudio-2022.07.1–554 was used for correlation analysis and graph construction.

### Chloroplast genome

#### DNA extraction

*I. indigotica* and *I. tinctoria* were selected at the same growth stage to extract genomic DNA (Fig. S1). Total DNA was extracted (kit: Dp360, Beijing Tiangen Biotechnology Co., Ltd, China) from 28 samples. The purified DNA was quantified spectrophotometrically with a microplate reader (Epoch2, BioTek, USA). The ratio of OD260/OD280 is a measure of the degree of protein contamination. For high–quality DNA, the OD260/OD280 reading is between 1.8 and 2.1, and a ratio of 2.0 is a sign of high-quality DNA.

#### Complete chloroplast genome annotation

The second-generation sequencing data for *I. indigotica* were downloaded from the NCBI (SRX10395440). GetOrganelle software (V1.7.3.4) was used to assemble and examine the chloroplast genome (*I. indigotica*). The *I. tinctoria* chloroplast genome was obtained from the NCBI (accession number NC_028415). The construction of the chloroplast genome was completed using software, and each base was manually determined to ensure its accuracy. The websites Cpgavas2 [11] and Geseq [12] were used to annotate genes of the chloroplast genome. All codons were manually adjusted in order, and the adjusted sequences were visualized by Organellar-GenomeDRAW [13], with default parameters set to construct a chloroplast genome map.

#### Comparative analyses of the chloroplast genomes of the two species

Geneious 11.1.2 was used to calculate the total length of the genome and each region (inverted repeat regions, small single-copy regions and large single-copy regions) in the two species. It also provided the base composition, GC (AT) content and gene composition of both genomes.

#### Codon analyses

Although codon usage bias occurs between species or during translation within the same species, some codon usage is high and needs to be fully analysed. As a measure reflecting the genetics and evolution of species, codon preference is frequently examined in similar studies [14]. In this work, we used CodonW to analyse codon preference.

#### Phylogenetic analysis

MAFFT (V7) was used for alignment, with default parameter settings [15]. IQ-TREE (V1.6.1) was used to construct a maximum likelihood (ML) tree [16]. This method is accurate, fast, flexible and extensively used to determine the genetic relationship between species. The best-fit model was found by ModelFinder, and the phylogenetic tree was reconstructed [17]. The best models for ITS2 (2 F/3R) and ITS2 (P3/E4) were TIM + F + R4 and TIM2 + F + R3, respectively. The best model for the mini-barcode was TIM2 + F + I. The ITS2 (2F3R) outgroup was *Nicotiana tabacum* (NCBI number: AJ012363). The sequence of *I. tinctoria* (NCBI number: FJ593182) was downloaded from the NCBI, and alignment analysis was performed. The outgroup of ITS2 (P3E4) was *Nicotiana glauca* (NCBI number: AJ012363). The *I. tinctoria* sequence (NCBI number: MT923072) was downloaded from the NCBI and compared for analysis. Phylogenetic tree reconstruction was based on the ML method with 1000 bootstrap replicates. iTOL software (https://itol.embl.de/) was used to display and annotate the phylogenetic tree.

#### Analysis of sequence divergences

Inverted Repeats Finder was used to detect IR regions, with default parameters. The length had to be at least 20 bp, and 90% sequence similarity was required [18]. To analyse differences in the chloroplast genome, we performed sliding window analysis using DnaSP (V6.11.0179) to evaluate nucleotide variability (PI) in the two species. Among the window parameter settings, the step size and length size were set to 200 and 600 bp, respectively [19]. According to the PI values, the highest point *rpl*32 ~ *trn*L–UAG was used for mini-barcode primer design to distinguish *I. indigotica* from *I. tinctoria.*

#### Validation of primers

PCR amplification was performed in a 20-µl total reaction, including 10 µl of CWBIO 2×Es MasterMix Dye, a 10-µM solution of mini-barcode primer (FR, 1 µl), approximately 7 µl of ddH_2_O, and 1 µl of DNA from 28 samples. The PCR program was as follows: 94 °C for 2 min, 94 °C for 30 s, 30 cycles including 58 °C for 30 s and 72 °C for 30 s, 72 °C for 2 min, and 4 °C to stop the reaction. The PCR amplicons were sequenced in both directions using the above primer (FR) on an AB 3730Xl DNA Sequencer (Applied Biosystems, U.S.A.) by Tsingke Biological Co., Ltd. The sequencing results were spliced using DNAMAN software (V10), and consistent sequences were obtained by bidirectional splicing for analysis.

### Genome survey of tetraploid *I. indigotica*

#### Extraction and sequencing of genomes

The CTAB method was used to extract genomic DNA from tetraploid *I. indigotica* (27). The MGI-2000 platform was used for sequencing at Shenzhen Huada Gene Technology Service Co., Ltd, and a 350 bp library was built. The amount of sequencing data was 50× for subsequent analysis.

#### Data filtering

SOAPnuke (V1.5.3) was used to filter the original sequencing data to remove low-quality reads, connectors and PCR duplicates [20]. The remaining clean reads were then used for subsequent analysis.

#### K-mer statistics and genome assessment

The reads obtained from sequencing were divided into K-mers, and statistical analysis of the genome and miscellaneous and duplicate records was performed. Jellyfish (V2.2.10) was used to quickly calculate the frequency of K-mers [21]. Genomescope software was fitted with the spectrum of the K-mer [22]. Smudgeplot software was used to determine the characteristics of the tetraploid *I. indigotica* genome.

## Results

### Morphological characteristics of the two species

**Fig. 1:**
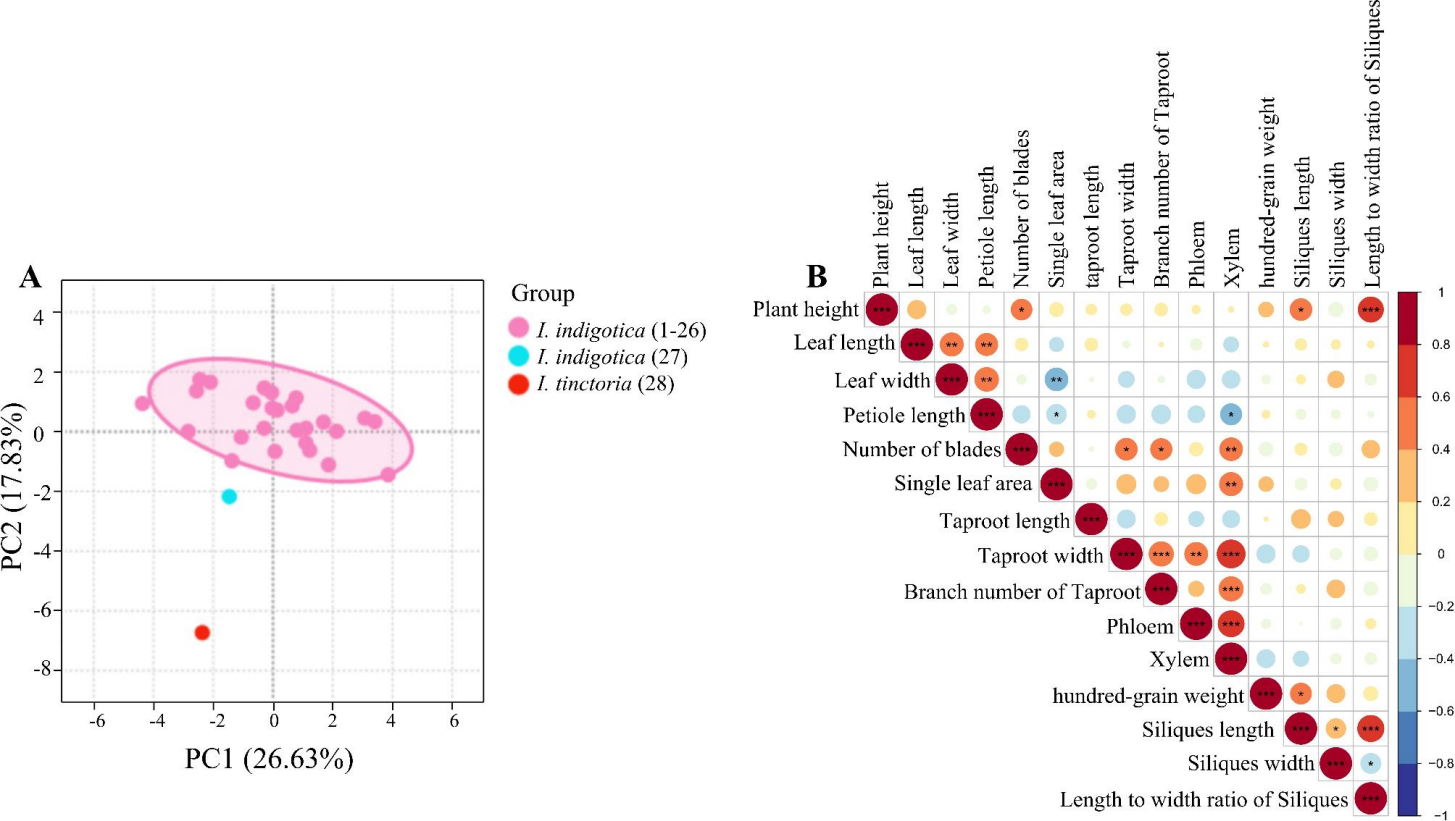
Analysis of differences in morphological data in 28 samples (**A:** PCA of morphological data of *I. indigotica* and *I. tinctoria*. **B:** Correlation analysis between morphological data of 28 samples. *P < 0.05, **P < 0.01, ***P < 0.001)

Morphological data of *I. indigotica* and *I. tinctoria* were recorded in different periods, and the results were analysed by PCA, as shown in Fig. 1A. All the samples of *I. indigotica* (1–26) were placed into one class, and tetraploid *I. indigotica* (27) and *I. tinctoria* (28) were placed into one class each. Specific phenotypic data are shown in Table S2. Most of the morphological data were significantly positively correlated, as shown in Fig. 1B, where the xylem and length to width ratio of siliques were significant (*P* < 0.001). The results shown in Fig. 1 indicate that the two species can be distinguished. However, because the morphological data were highly inconsistent, they did not provide strong evidence, and further verification is needed at the molecular level.

### Complete chloroplast genome features of the two species

**Fig. 2:**
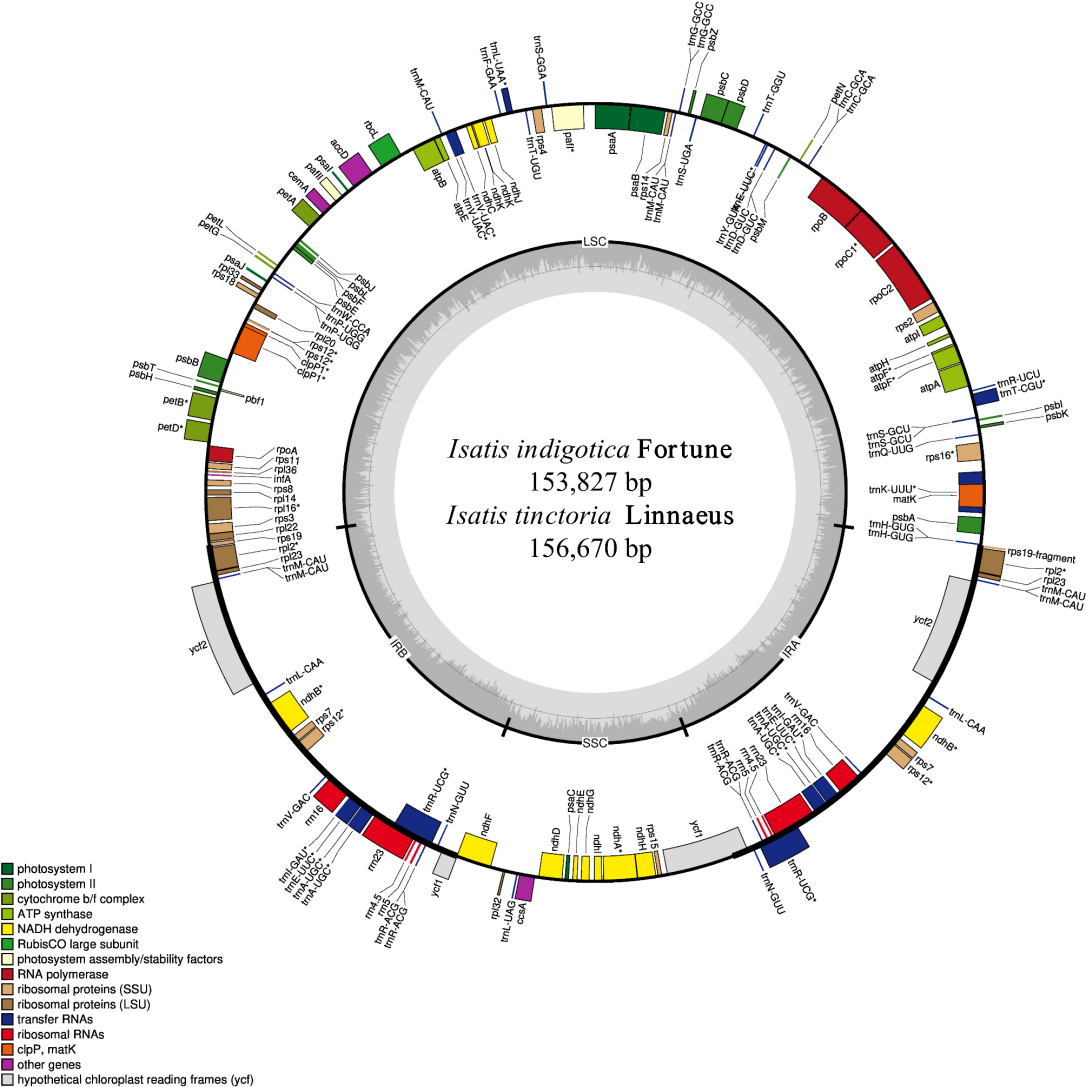
Gene map of the complete chloroplast genomes of the two *Isatis* species. Genes on the inside of the circle are transcribed clockwise, whereas those outside are transcribed counterclockwise. The light grey and dark grey shading within the inner circle correspond to the percentages of A + T and G + C contents, respectively

The two species displayed a similar tetrad structure as shown in Fig. 2. The chloroplast genome sizes of *I. indigotica* and *I. tinctoria* were 153,827 bp, and 156,670 bp, respectively. There were 132 genes, including 8 rRNA genes, 37 tRNAs, and 87 protein coding genes. *I. indigotica* included a pair of IR regions of 26,272 bp each, separated by an LSC region of 83,577 bp, and an SSC region of 17,706 bp. *I. tinctoria* had a pair of IR regions of 26,995 bp each, separated by a LSC region of 84,907 bp, and a SSC region of 17,773 bp. The total GC content was 36.5%, while the corresponding values of the LSC, SSC and IR regions were 34.2%, 29.7% and 42.3%, respectively (Table 1). The nucleotide composition of the chloroplast genomes of the two species was biased towards T and A. The two species had the same LSC region, and each region contained ATCG (Table 2).

**Table 1:** Comparison of the chloroplast genome organization of the two *Isatis* species

Genome features	*Isatis indigotica* Fortune	*Isatis tinctoria* Linnaeus
Size (bp)	153,827	156,670
LSC (bp)	83,577	84,907
SSC (bp)	17,706	17,773
IR (bp)	26,272	26,995
Total number of genes	131	131
Protein-coding genes	87	87
tRNA genes	37	37
rRNA genes	8	8
Total G + C content (%)	36.5	36.5

**Table 2:** Base composition in a single strand of the cp. genome of the two species

Species	Region	A (%)	C (%)	G (%)	T (%)	AT (%)	GC (%)
*I. indigotica*	LSC	32.0	17.5	16.7	33.8	65.8	34.2
	SSC	35.2	15.3	14.3	35.1	70.4	29.6
	IRa	28.9	22.0	20.3	28.8	57.7	42.3
	IRb	28.8	20.3	22.0	28.9	57.7	42.3
*I. tinctoria*	LSC	32.0	17.5	16.7	33.7	65.8	34.2
	SSC	35.1	15.3	14.4	35.2	70.4	29.6
	IRa	29.0	22.0	20.4	28.7	57.7	42.3
	IRb	28.7	20.4	22.0	29.0	57.7	42.3

LSC (Long Single-Copy sequence). SSC (Short single-copy sequence). IRa and IRb (Reverse Repeat sequences, same coding, opposite direction)

Table 3 shows related genes, including self–replicating genes, genes involved in photosynthesis, genes with unknown functions and other genes in the two species [19]. Fifteen genes (*rpl*2, *rpl*16, *rps*16, *rpo*C1, *trn*A–UGC, *trn*E–UCC, *trn*K–UUU, *trn*L–UAA, *trn*V–UAC, *trn*T–UGU, *ndh*A, *ndh*B, *pet*B, *pet*D, and *atp*F) each contained one intron, and two genes, i.e., *clp*P and *ycf*3, harboured two introns each.

**Table 3:** Gene contents in the chloroplast genomes of the two *Isatis* species

Gene category	Gene groups	Names of genes
Self-replicating	Large subunit of ribosome (LSU)	*rpl*14, *rpl*16_a_, *rpl*2_a_ (2), *rpl*20, *rpl*22, *rpl*23 (2), *rpl*32, *rpl*33, *rpl*36
	Small subunit of ribosome (SSU)	*rps*11, *rps*12_c_ (2), *rps*14, *rps*15, *rps*16_a_, *rps*18, *rps*19 (2), *rps*2, *rps*3, *rps*4, *rps*7 (2), *rps*8
	DNA dependent RNA polymerase	*rpo*A, *rpo*B, *rpo*C1_a_, *rpo*C2
	rRNA genes	*rrn*16 (2), *rrn*23 (2), *rrn*4.5 (2), *rrn*5 (2)
	tRNA genes	*trn*A-UGC_a_ (2), *trn*C-GCA, *trn*D-GUC, *trn*E-UUC_a_ (2), *trn*F- GAA, *trn*G-GCC, *trn*H-GUG, *trn*P-UGG,*trn*K-UUU_a_, *trn*L-CAA (2), *trn*L-UAA_a_, *trn*L-UAG, *trn*M-CAU (4), *trn*S-GCU, *trn*S-GGA, *trn*S-UGA, *trn*T-CGU_a_, *trn*T-UGU, *trn*V-GAC (2), *trn*V-UAC_a_, *trn*W-CCA, *trn*Y-GUA, *trn*Q-UUG, *trn*R-ACG (2), *trn*R-UCU (2)
Photosynthesis	Photosystem I	*psa*A, *psa*B, *psa*C, *psa*I, *psa*J
	Photosystem II	*psb*A, *psb*B, *psb*C, *psb*D, *psb*E, *psb*F, *psb*G, *psb*H, *psb*I, *psb*J, *psb*K, *psb*L, *psb*M, *psb*N, *psb*T, *psb*Z
	NADH dehydrogenase	*ndh*A_a_, *ndh*B_a_ (2), *ndh*C, *ndh*D, *ndh*E, *ndh*F, *ndh*G, *ndh*H, *ndh*I, *ndh*J
	Cytochrome b/f complex	*pet*A, *pet*B_a_, *pet*D_a_, *pet*G, *pet*L (2)
	Subunits of ATP synthase	*atp*A, *atp*B, *atp*E, *atp*F_a_ (2), *atp*H
	Large subunit of Rubisco	*rbc*L
Other genes	Protease	*clp*P_b_
	Maturase	*mat*K
	Envelop membrane protein	*cem*A
	Subunit of acetyl-CoA	*acc*D
Unknown function	Proteins of unknown function	*ycf*1 (2), *ycf*2 (2), *ycf*3_b_, *ycf*4, *ycf*5, *ycf*6, *ycf*15 (2)

^a^ Single intron

^b^ Genes containing two introns

^c^ Genes divided into two independent transcription units

### Codon usage analyses

**Fig. 3:**
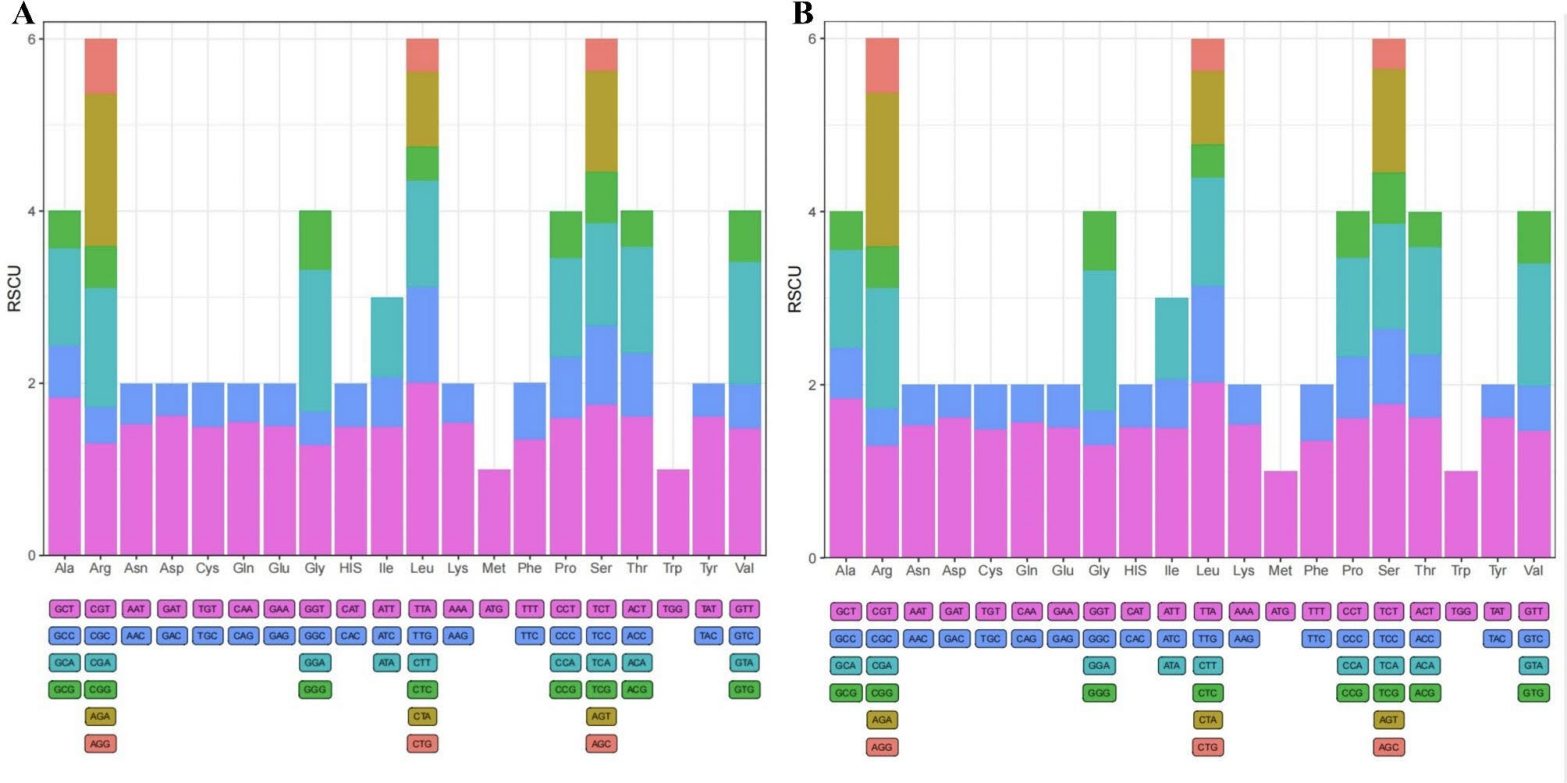
Codon content of amino acids encoding proteins in the chloroplast genome of the two species (**A:***I. indigotica.***B:***I. tinctoria*)

The usage frequency of 20 amino acids was between 1.63% and 9.38%, as shown in Fig. 3. Arg, Leu and Ser were the most abundant amino acids, and the most commonly used codons were AGA, TTA, and TCT. Met and Trp were represented by only one codon, ATG and TGG, respectively, and other amino acids were encoded by 2–5 amino acids. Four synonymous codons were used for Ala, Gly, Pro, Thr, and Val, and the most commonly used codons were GCT, GCA, CCT, ACT and GTA. Three synonymous codons was observed only for IIe, and the most commonly used codons was ATT. Asn, Asp, Cys, Glu, Gln, His, Lys, Phe, and Tyr, which were used at different frequencies, were encoded by AAT, GAT, TGT, GAA, CAA, CAT, AAA, TTT and TAT, respectively. In the two species, 35 codons had a RSCU > 1, of which 30 codons ended in A/T, accounting for 85.71%. Twenty-six codons had RSCU < 1, all of which ended in CG.

### Analysis of inverted repeats and barcoding

**Fig. 4:**
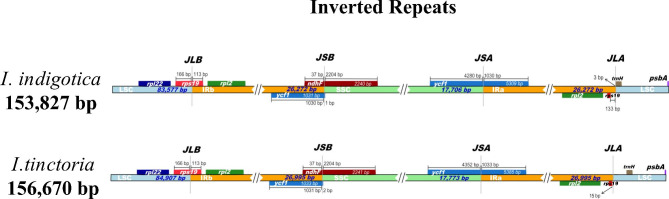
Comparison of chloroplast genome genome sequences of *I. indigotica* and *I. tinctoria* at the junction of the LSC, LSC, IR (IRa and IRb), and SSC regions

Although chloroplast genomes are highly conserved in terms of structure and size, expansion and contraction of the LSC/IRb, IRb/SSC, SSC/IRa, and IRa/SSC boundaries can lead to changes in the length of the junction region, in turn causing changes in the chloroplast genome [24]. The *rps*19 gene was located at the junction of LSC/IRb; the *ndh*F and *ycf*1 genes were located at the junction of IRb/SSC, and most of the *ndh*F gene extended to the SSC region. The *ycf*1 gene was located at the SSC/IRa junction, and most of it extended to the SSC region. The *trn*H gene was located at the IRa/SSC junction, where the distance between *trn*H and the JLA of *I. indigotica* was 3 bp, and the *rpl*2 gene was slightly contracted towards the IRa region. Fig. 4 shows that the IR/SC junction has almost the same length, with only slight dilation and contraction, indicating that the chloroplast genomes of the two species are highly similar.

**Fig. 5:**
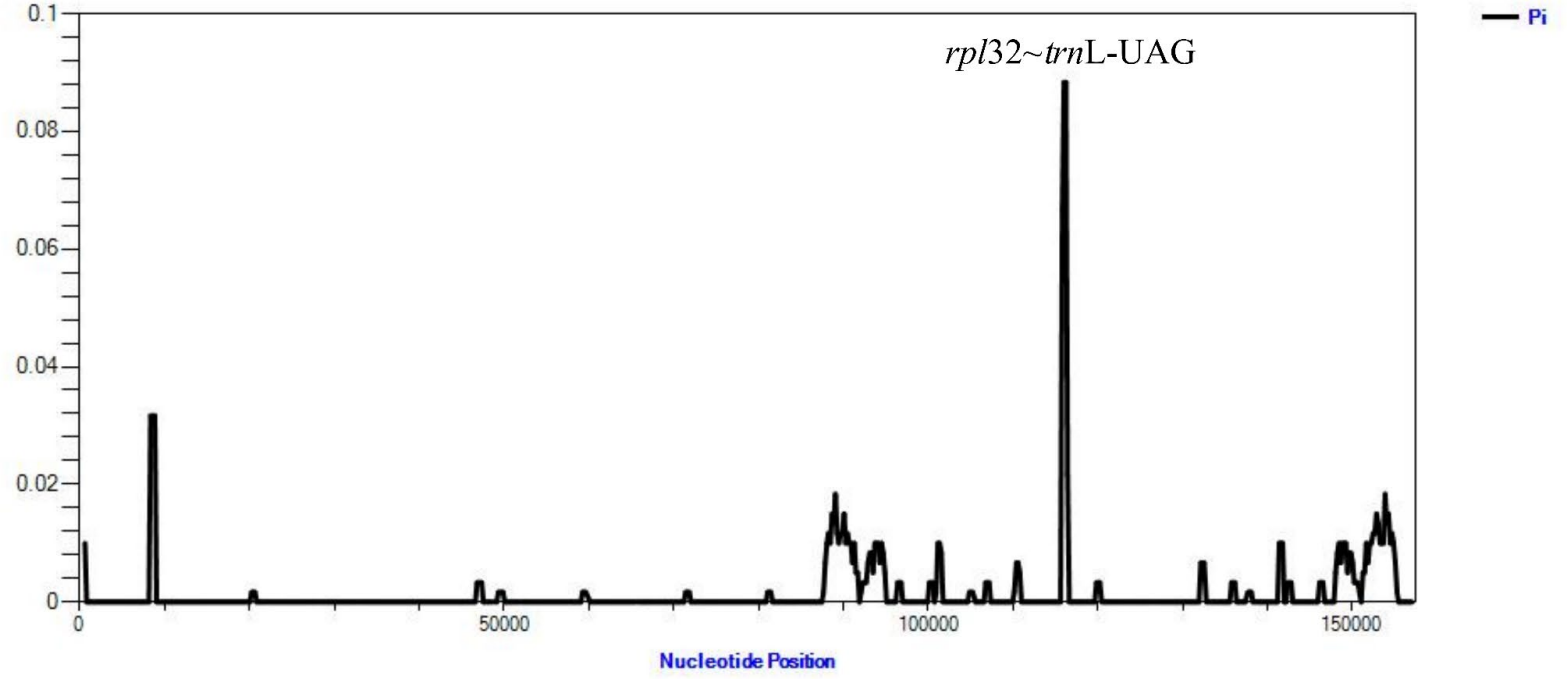
Nucleotide diversity (Pi) based on sliding window analysis of *I. indigotica* and *I. tinctoria* chloroplast genomes

Traditional barcoding was performed using the nuclear genome sequence ITS2 (2 F: ATGCGATACTTGGTGTGAAT, 3R: GACGCTTCTCCAGACTACAAT; P3: YGACTCTCGGCAACGGATA, E4: RGTTTCTTTTCCTCCGCTTA). Due to the highly variable DNA regions of the chloroplast genome, researchers have attempted to look for differences in these genomes to differentiate species [23]. The chloroplast genomes of two *Isatis* species, including 132 genes, were analysed to estimate nucleotide diversity (Pi). Fig. 5 shows that the mean value of nucleotide variability for the two species is 0.00161, ranging from 0.00001 to 0.08333. We found that the *rpl*32 ~ *trn*L–UAG region showed a higher Pi value (0.08333), and thus it was considered a candidate marker to distinguish these two species. Primer design for the *rpl*32 ~ *trn*L–UAG region was performed using primer3 (F:ACCTTGATGCAATAAT.AAACAAAGA, R: AAAATGAAAACTTCTCCAAAATGC).

### Phylogenetic analysis

**Fig. 6:**
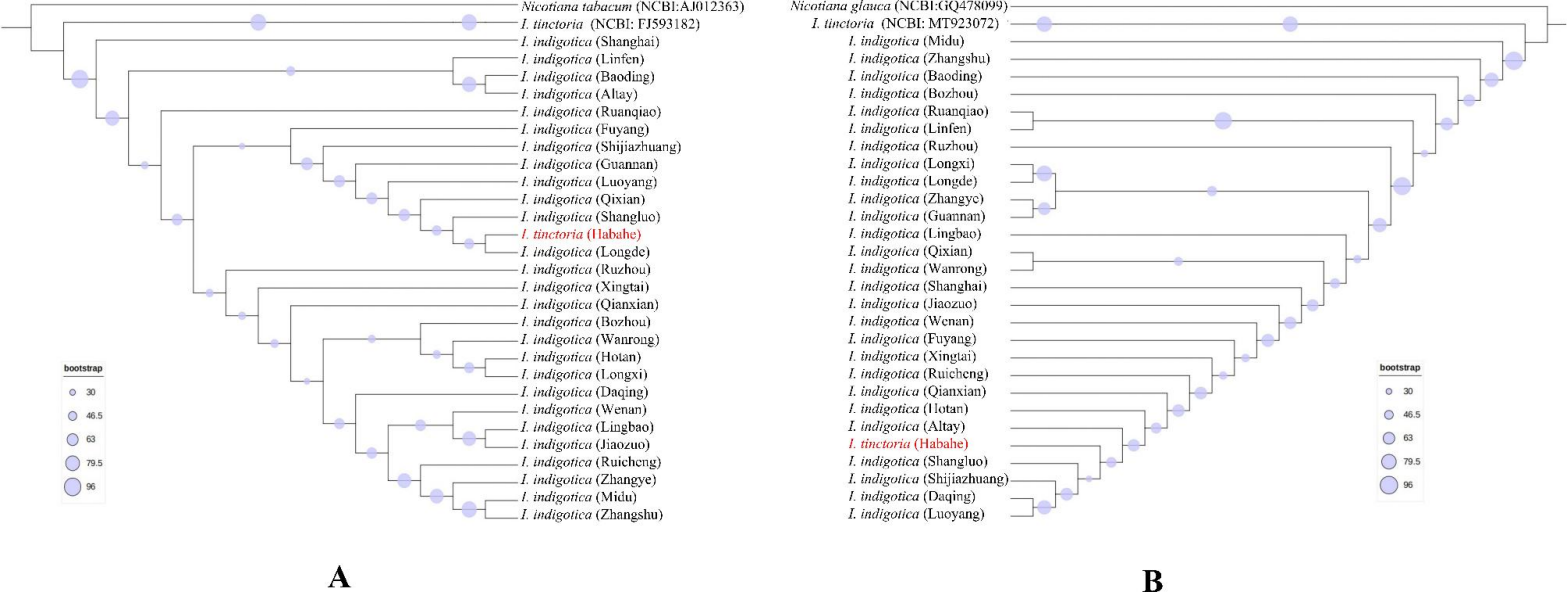
Maximum likelihood (ML) phylogenetic tree reconstruction based on ITS2 (circle size at the nodes on the tree represents the bootstrap value). [**A:** ITS2 with 2 F/3R primers. **B:** ITS2 with P3/E4 primers]

**Fig. 7:**
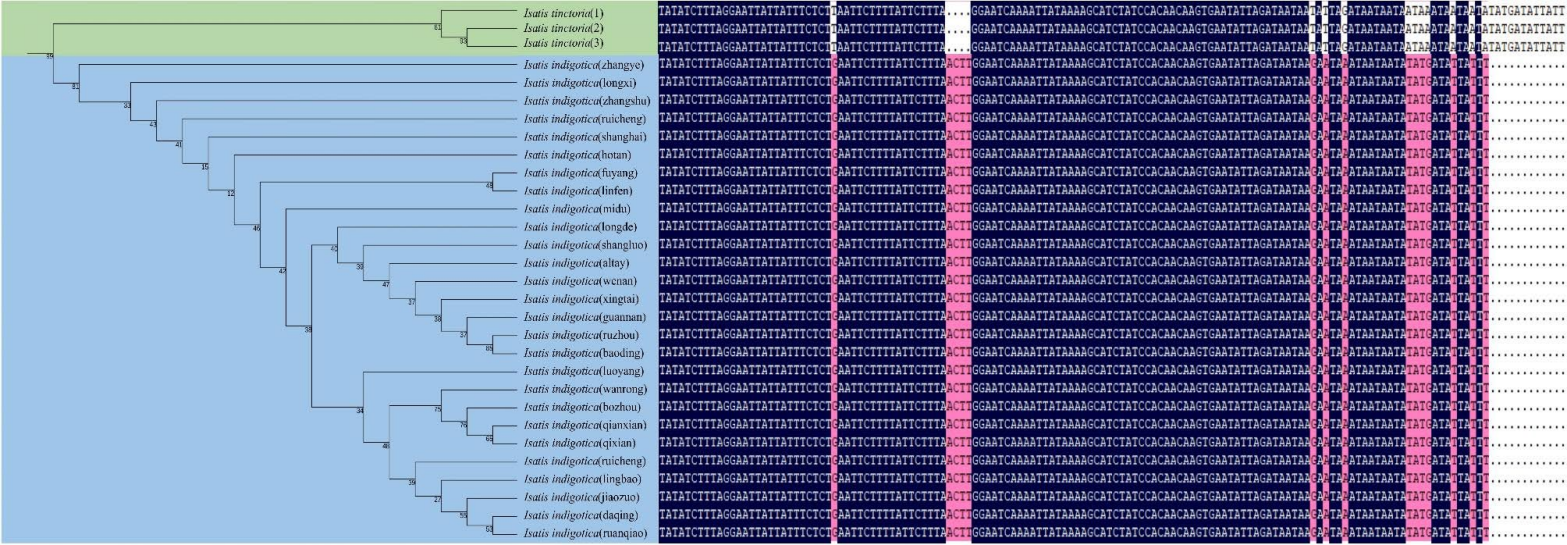
Maximum likelihood (ML) phylogenetic tree reconstruction based on the mini-barcode (numbers at the nodes on the tree represent bootstrap values) and differences in sequence and ploidy between *I. indigotica* and *I. tinctoria*

In recent years, as the most advanced method, high-throughput sequencing has provided convincing data for species identification and assisted in the establishment of phylogenies [24]. In Fig. 6, despite the addition of NCBI sequences of *I. tinctoria* (NCBI numbers: FJ593182 and AJ012363), *I. tinctoria* (Habahe) was still mixed with *I. indigotica.* This indicates that ITS2 (2F3R/P3E4) cannot be used to distinguish between the two species. However, the chloroplast genome, as an indicator of the phylogenetic relationships of inferred plant groups, is reliable. According to Fig. 7, there were 28 samples of *I. indigotica* and *I. tinctoria* (explant 1 and sterile seedlings cultured on MS media 2 and 3). The mini-barcode ML tree could accurately identify two species with high statistical support (bootstraps of 99%). Using DNAMAN to align the sequences of *rpl*32 ~ *trn*L–UAG in 28 samples, the sequences of diploid and tetraploid *I. indigotica* (1–27) were consistent. The sequences of *I. indigotica* (1–27) and *I. tinctoria* (28) were markedly different, and base mismatches and deletions were present. The mini-barcode can effectively distinguish *I. indigotica* from *I. tinctoria*.

### Filtering data volume and statistics

As shown in Table 4, filtering of raw sequencing data was performed by SOAPnuke (V1.5.3). The sample insert size was 300–400 bp, the length was 150 bp, the amount of clean data was 61.58 GB, and the GC content was 38.88%. The samples were thus qualified for subsequent experimental analysis.

**Table 4:** Filter data statistics

Lip ID	Insert Size /bp	Read Length /bp	Clean Data /Gb	GC /%
*I. Indigotica*(27)	300–400	150	61.58	38.88

### K-mer analysis

**Fig. 8:**
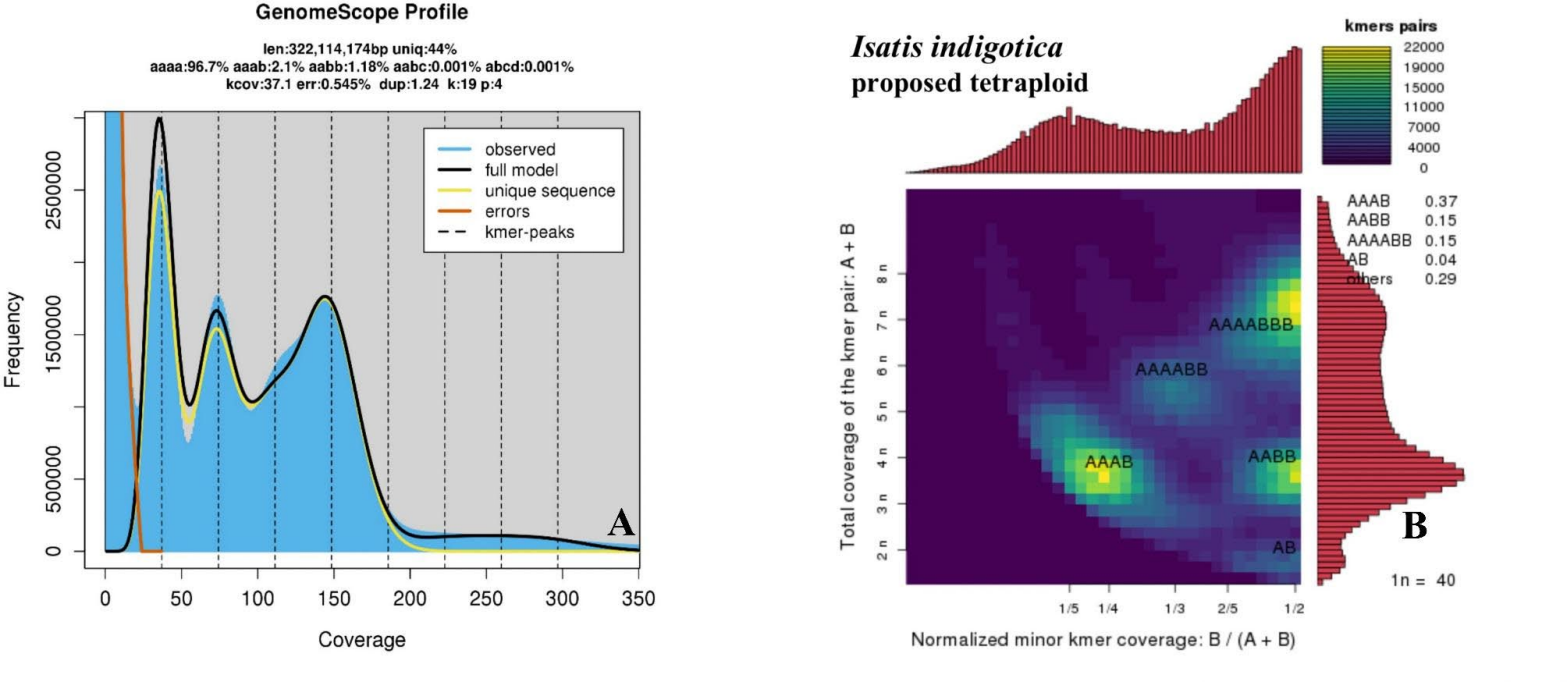
Genomic assessment of tetraploid *I. indigotica.* [**A**:19 K-mer distribution curve from GenomeScope 2 (the abscissa is the 19 K-mer depth (coverage), and the ordinate is the 19 K-mer frequency at this depth). **B**. Genomic ploidy evaluation of the smudgeplot

K-mers of 17–31 units were selected for statistical analysis, and the frequency of the 17–31-mers was quickly calculated using Jellyfish software (V2.2.10). The results are shown in Table 5, and the statistics are as follows. A negative binomial distribution model (Genomescope) was used, and 19 K-mer classes were selected for statistical analysis. The results demonstrated that the genome size was 643.77 MB, the heterozygosity rate was approximately 0.98%, the duplication rate was 90.43%, the fault tolerance rate was 0.55%, and the effective sequencing depth was 95.13 times. Fig. 8 A shows the size, depth, and predicted ploidy of the genome, indicating that aaaa was 96.7%, aabb was 1.18%, aabc was 0.001%, and abcd was 0.001%.

**Table 5:** K-mer analysis data statistics

K-mer	nkmer	Used Base	Genome Size	Heterozygosity Rate /%	Repeat Rate /%	Err Rate /%	Depth
17	53,851,163,098	61,269,199,597	640,214,171	1.02	92.16	0.51	95.70
19	53,158,325,118	61,244,074,835	643,773,428	0.98	90.43	0.55	95.13
21	52,449,263,250	61,245,289,199	645,628,445	0.93	89.44	0.54	94.86
23	51,729,586,094	61,251,901,691	647,066,203	0.88	88.68	0.53	94.66
25	51,000,392,123	61,262,821,109	648,564,597	0.83	88.02	0.52	94.46
27	50,261,501,940	61,269,593,710	649,904,178	0.79	87.41	0.50	94.27
29	49,513,713,275	61,276,033,162	651,165,210	0.76	86.85	0.49	94.10

Genome ploidy was assessed using a smudgeplot, and the results are shown in Fig. 8B. K-mer pairs from 0 to 22,000 were used to examine the genome, and it was found that the genotype frequencies were AAAB 0.37, AABB 0.15, AAAABB 0.15, AB 0.14, and others 0.29. Based on the results in Fig. 8AB, it was found that the species is autotetraploid and has a highly heterozygous and ultra-repetitive genome.

## Discussion

### Mini barcode effectively distinguishes *I. indigotica* from *I. tinctoria*

With technological development, the methods of plant classification and identification are constantly updated, and plants are classified on the basis of morphological characteristics, tissue structure, ploidy analysis and genetic composition. Generally, ploidy analysis is performed on chromosomes and DNA content [4] to compensate for the lack of morphological and tissue structure information for identification. *I. indigotica* is mostly cultivated in various parts of China, and may be diploid or tetraploid (chromosome number of 2n = 14 and 2n = 28, respectively). Most *I. tinctoria* plants are wild and tetraploid (the number of chromosomes is 2n = 28) [1, 25]. Therefore, tetraploid *I. indigotica* and *I. tinctoria* cannot be distinguished by ploidy analysis. Amplification using standard, variable, easily amplified, relatively short DNA fragments, known as DNA barcoding, is emerging as a new biometric system [26]. In contrast to morphology, it can support classification basis at the gene level, which can compensate for the lack of identification information due to the similarity in chromosomal ploidy. Although the above methods are effective, they may not be able to accurately identify species with highly similar genotypes.

In this work, it was found that the chloroplast genomes of *I. indigotica* and *I. tinctoria* were 153,827 bp and 156,670 bp long, respectively, and they encoded a total of 132 genes. The chloroplast genomes of these two species are highly conserved and similar, It may not be possible to accurately distinguish using traditional barcoding techniques. This work confirmed this speculation; ITS2 (2F3R/P3E4) could not be used to distinguish between *I. indigotica* and *I. tinctoria.* The species were relocated to a branch on the NJ tree in Fig. 6. Given that the above techniques are the most advanced among plant classification techniques, *FOC* treating *I. indigotica* and *I. tinctoria* as one species seems to be correct. The mini-barcode showed different results. In the phylogenetic analysis, *I. tinctoria* formed a monophyletic clade with high bootstrap support separate from *I. indigotica*. On the phylogenetic tree, diploid and tetraploid *I. indigotica* belonged to a single DNA–barcode category that was different from that of *I. tinctoria. I. indigotica* and *I. tinctoria* are clearly not the same species because of their different ploidies and different genes. Due to the different evolutionary rules of the chloroplast genome and nuclear genome sequences, where the former is biparentally inherited [27] and the latter is maternally inherited [28], it is necessary to take into account both the chloroplast genome and nuclear genome for species identification.

Despite the versatility of the molecular markers developed, they have not been adequately tested to identify the homology of polyploid *I. indigotica.* However, they can distinguish between *I. indigotica* and *I. tinctoria*. The findings of this study are consistent with research results reported by Chinese scholars [6] as well as some European scholars who have ensured that they are two independent species based on chemical composition and molecular markers [29]. However, their merging in the *FOC* is not supported. This method will be used to guide the relevant quality control research on other Chinese herbal medicines, especially adulterants and counterfeits, and will be continuously applied in related research fields.

### The source of tetraploid *I. indigotica*

Polyploidy is a natural phenomenon of plant evolution and an important evolutionary process in adaptation to the natural environment [30]. It is also an important step in the formation of new species. Angiosperms are mostly polyploid, with more than approximately 70% including many important crops, such as *Brassica napus* [31], *Triticum aestivum* [32] and *Gossypium spp* [33]. Compared with diploid plants, polyploid plants are large and have the advantages of enhanced resistance, improved quality and increased yield [34].Autopolyploidy refers to the phenomenon in which the chromosomes of an organism come from different species and are generally produced by the direct doubling of diploid chromosomes [35]. Allopolyploidy refers to chromosomes in an organism from different species, which is the product of hybridization doubling of different species or autopolyploid hybridization of different species [36]. *I. indigotica* is not highly fertile due to self-incompatibility [37]; therefore, it is important to clarify the source of germplasm [38].

The use of autopolyploid plant breeding can increase the content of secondary metabolites and active ingredients [39]. The active components in the leaves and roots of tetraploid *I. indigotica* were more abundant than those in diploid *I. indigotica* [39]. Tetraploid *I. indigotica* exhibited greater resistance and higher yields, while being more adaptable [40]. The above results do not indicate the source of polyploid *I. indigotica* as a wild-type or artificially doubled polyploid. In this work, the K-mers in the tetraploid *I. indigotica* genome sequence were analysed, and Genomescope analysis and a smudgeplot were combined to find that the genome was highly heterozygous and extremely repetitive, and the species was revealed to be an artificially doubled polyploid, not an allopolyploid.

Given that genome sequencing has become the most effective tool for species identification and assessment [41], the technique can be used as a reference for the identification of polyploid genomes, through which it can be applied to other complex genomes, not to mention highly similar homologous genomes, to identify their origin [42]. This approach makes up for the shortcomings of mini-barcodes, taking into account the chloroplast genome and high-throughput sequencing technology to completely identify them, and reveals the source of tetraploid *I. indigotica*. This study provides molecular evidence for the identification of the original species of Isatidis Radix and Isatidis Folium used as traditional medicinal materials in China.

## Conclusions

In this study, we successfully used a mini-barcode to distinguish *I. indigotica* and *I. tinctoria*. The mini–barcode primers *rpl*32 ~ *trn*L–UAG could accurately identify these two species. Tetraploid *I. indigotica* is an autotetraploid, and this species has a highly heterozygous and extremely repetitive genome, which can be used to analyse the evolutionary relationships within *I. indigotica.* Mini-barcoding can help to further elucidate the genetic diversity of *I. indigotica* and *I. tinctoria*. It can provide a reference for phylogenetic and evolutionary studies.

### Electronic supplementary material

Below is the link to the electronic supplementary material.

Supplementary Material 1:
**Figure S1**. Morphological Characteristics of *I. indigotica* and *I. tinctoria*.
** Table S1**. The information of the seeds.
** Table S2**. Morphological data in *I. indigotica* and *I. tinctoria*.
** Sequence of ITS2-2F(1~28).**
** Sequence of ITS2-p3(1~28).**
** Sequence of mini-barcode(1~28)**

## Data Availability

The chloroplast genome of *I. indigotica* has been uploaded to the NCBI database (GenBank accession number OP620952) and the chloroplast genome fasta, gb and sqn files were uploaded to Figshare: https://figshare.com/articles/thesis/genbank_Isatis_ indigotica_Fortune_sqn/21,371,487. The chloroplast genome of *I. tinctoria* was obtained from the NCBI (accession number NC_028415). The de novo clean reads of tetraploid *I. indigotica* were uploaded to the NCBI SRA database under BioProject PRJNA888437.
