# Supplementary material for: Classification of Isatis indigotica Fortune and Isatis tinctoria Linnaeus via comparative analysis of chloroplast genomes
Source: BMC Genomics. 2023 Aug 18;24:465. doi: 10.1186/s12864-023-09534-8 (PMC10436401; doi:10.1186/s12864-023-09534-8)
Supplement: Supplementary file 1 — Supplementary Material 1: Figure S1. Morphological Characteristics of I. indigotica and I. tinctoria. Table S1. The information of the seeds. Table S2. Morphological data in I. indigotica and I. tinctoria. Sequence of ITS2-2F(1~28). Sequence of ITS2-p3(1~28). Sequence of mini-barcode(1~28) [file 12864_2023_9534_MOESM1_ESM.docx]

**Figure S1.** Morphological Characteristics of *I. indigotica* and *I. tinctoria*.

**Table S1.** The information of the seeds.

**Table S2.** Morphological data in *I. indigotica* and *I. tinctoria.*

**Figure S1**. Morphological Characteristics of *I. indigotica* and *I. tinctoria*. Bar=1 cm.

A: The seeds of *I. indigotica* (Altay Prefecture, Xinjiang),

B: The seeds of *I. tinctoria* (Habahe, Xinjiang),

C: The seeds of *I. indigotica* (Qianxian, Shanxi),

D: Seedlings of *I. indigotica* (Altay Prefecture, Xinjiang; 14 d),

E: Seedling of *I. tinctoria* (Habahe, Xinjiang; 14 d),

F: Seedling of *I. indigotica* (Qianxian, Shanxi; 14 d).


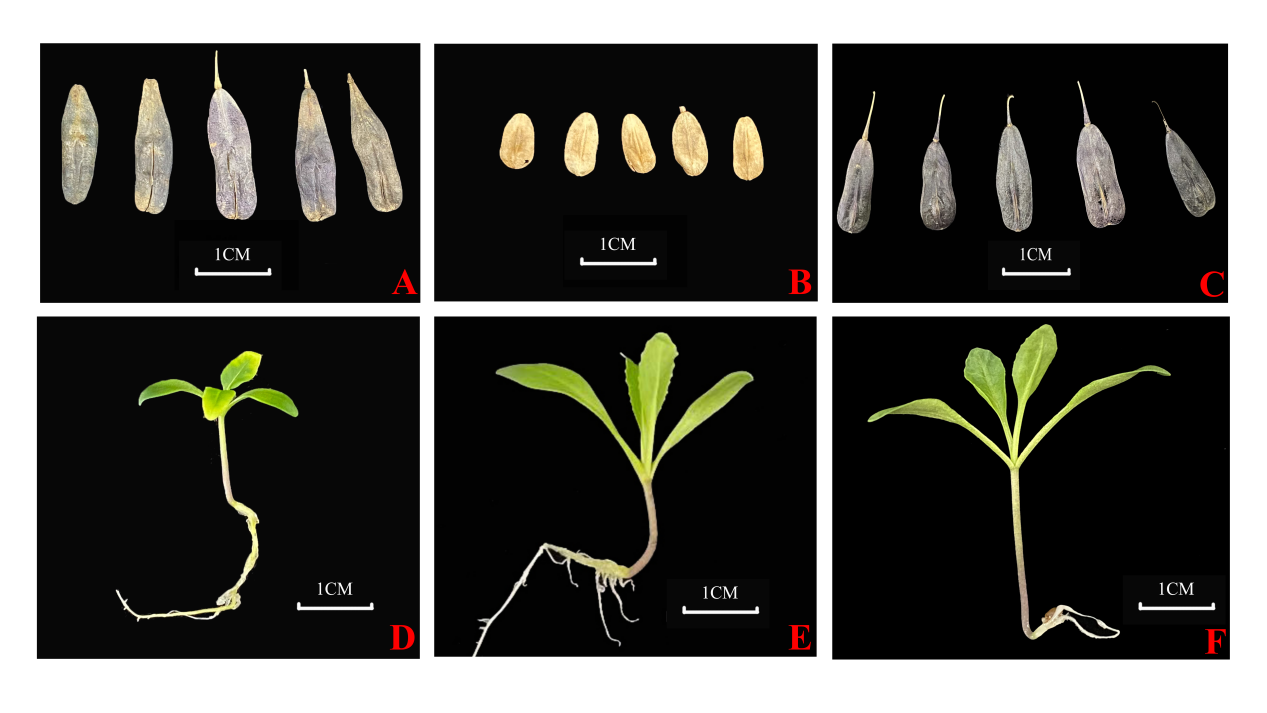


**Table S1** The information of the seeds

| **No.^a^** | **Species** | **Location** | **Origin** |
| --- | --- | --- | --- |
| 1 | *I. indigotica* | N35°42', E110°84' | Wanrong, Shanxi, China |
| 2 | *I. indigotica*(blank) | N43°80', E87°63' | Altay Prefecture, Xinjiang, China |
| 3 | *I. indigotica* | N34°70', E110°70' | Ruicheng, Shanxi, China |
| 4 | *I. indigotica* | N36°09', E111°53' | Linfen, Shanxi, China |
| 5 | *I. indigotica* | N33°37', E115°74' | Ruanqiao, Anhui, China |
| 6 | *I. indigotica* | N33°85', E115°78' | Bozhou, Anhui, China |
| 7 | *I. indigotica* | N38°88', E115°47' | Baoding,Hebei, China |
| 8 | *I. indigotica* | N34°09', E119°32' | Guannan, Jiangsu, China |
| 9 | *I. indigotica* | N34°62', E112°46' | Luoyang, HeNan, China |
| 10 | *I. indigotica* | N35°22', E113°25' | Jiaozuo, Henan, China |
| 11 | *I. indigotica* | N34°55', E114°79' | Qixian, Henan, China |
| 12 | *I. indigotica* | N34°17', E112°85' | Ruzhou, Henan, China |
| 13 | *I. indigotica* | N34°52', E110°90' | Lingbao, Henan, China |
| 14 | *I. indigotica* | N38°88', E116°46' | Wenan, Hebei, China |
| 15 | *I. indigotica* | N38°05', E114°52' | Shijiazhuang, Hebei, China |
| 16 | *I. indigotica* | N33°88', E109°92' | Shangluo, Shanxi, China |
| 17 | *I. indigotica* | N35°63', E106°12' | Longde, Ningxia, China |
| 18 | *I. indigotica* | N38°93', E100°46' | Zhangye, Gansu, China |
| 19 | *I. indigotica* | N35°01', E104°64' | Longxi, Gansu, China |
| 20 | *I. indigotica* | N28°06', E115°55' | Zhangshu, Jiangxi, China |
| 21 | *I. indigotica* | N25°35', E100°50' | Midu, Yunnan, China |
| 22 | *I. indigotica* | N37°12', E79°94' | Hotan, Xinjiang, China |
| 23 | *I. indigotica* | N32°90', E115°82' | Fuyang, Anhui, China |
| 24 | *I. indigotica* | N46°59', E125°11' | Daqing, Heilongjiang, China |
| 25 | *I. indigotica* | N37°08', E114°51' | Xingtai, Hebei, China |
| 26 | *I. indigotica* | N31°24', E121°48' | Shanghai, China |
| 27 | *I. indigotica* | N34°53', E108°24' | Qianxian, Shaanxi, China |
| 28 | *I. tinctoria* | N48°06', E86°42' | Habahe, Xinjiang, China |

^a^Seed Number

**Table S2** Morphological data in *I. indigotica* and *I. tinctoria* (N=10)

| No.^a^ | Plant height  /cm | Leaf length  /cm | Leaf width  /cm | Petiole length  /cm | Number of blades  /piece | Single leaf area  /cm^2^ | Taproot length  /cm | Taproot width  /mm | Branch number of Taproot  /piece | Phloem  /mm | Xylem  /mm | hundred-grain weight  /g | Siliques length  /cm | Siliques width  /cm | Length to width ratio of Siliques |
| --- | --- | --- | --- | --- | --- | --- | --- | --- | --- | --- | --- | --- | --- | --- | --- |
| 1 | 27.40±0.7 | 26.10±1.42 | 5.17±0.61 | 4.77±0.25 | 32.00±4.24 | 52.76±13.73 | 9.00±0 | 1.40±0 | 5.00±0 | 0.96±0.11 | 3.83±0.45 | 0.89±0.07 | 1.71±0.03 | 0.40±0.02 | 4.32±0.03 |
| 2 | 27.13±2.72 | 28.50±3.59 | 4.80±0.17 | 4.17±1.53 | 24.67±6.43 | 60.91±8.49 | 9.00±1.41 | 1.40±0.28 | 7.00±1.41 | 0.77±0.06 | 3.45±0.23 | 0.93±0.03 | 1.80±0.18 | 0.40±0.06 | 4.51±0.12 |
| 3 | 26.97±0.32 | 26.03±2.50 | 5.97±0.64 | 3.07±0.38 | 29.33±6.43 | 36.61±3.18 | 18.50±0 | 1.50±0 | 18.00±0 | 0.64±0.13 | 3.82±0.37 | 0.77±0.02 | 1.91±0.13 | 0.50±0.01 | 3.79±0.07 |
| 4 | 19.50±0.36 | 23.87±1.17 | 6.80±0.46 | 7.63±0.91 | 7.00±1.41 | 26.87±6.81 | 16.50±0 | 0.50±0 | 4.00±0 | 0.35±0.05 | 1.45±0.18 | 0.99±0.06 | 1.84±0.11 | 0.49±0.02 | 3.76±0.06 |
| 5 | 25.53±0.25 | 37.57±0.97 | 6.67±0.83 | 10.43±1.48 | 14.50±3.54 | 32.45±8.20 | 16.00±2.00 | 0.87±0.15 | 4.33±1.53 | 0.76±0.24 | 1.76±0.44 | 1.05±0.06 | 1.81±0.16 | 0.48±0.03 | 3.77±0.14 |
| 6 | 25.90±0.44 | 27.67±2.48 | 6.70±0.80 | 9.67±1.96 | 11.67±1.53 | 27.50±9.79 | 5.17±0.29 | 0.83±0.12 | 5.33±1.15 | 0.74±0.11 | 2.21±0.06 | 0.88±0.04 | 1.92±0.13 | 0.46±0.07 | 4.17±0.10 |
| 7 | 21.67±0.45 | 20.63±1.14 | 3.87±0.06 | 4.17±0.68 | 12.00±2.00 | 47.68±11.72 | 22.25±1.77 | 0.85±0.21 | 7.5±0.71 | 0.72±0.14 | 2.63±0.21 | 1.02±0.13 | 2.03±0.30 | 0.50±0.06 | 4.06±0.18 |
| 8 | 25.03±0.32 | 29.17±1.86 | 5.87±1.03 | 9.03±0.98 | 17.33±0.58 | 57.19±16.22 | 11.33±2.02 | 1.20±0.30 | 9.33±3.21 | 0.49±0.07 | 2.99±0.10 | 1.16±0.03 | 1.57±0.05 | 0.45±0.04 | 3.48±0.04 |
| 9 | 22.27±0.21 | 29.53±0.78 | 5.97±0.35 | 4.70±1.10 | 19.67±0.58 | 38.28±12.57 | 11.00±6.06 | 0.95±0.21 | 7.00±1.00 | 1.00±0.06 | 2.14±0.03 | 1.13±0.06 | 2.09±0.20 | 0.53±0.04 | 3.94±0.03 |
| 10 | 22.20±0.61 | 26.77±1.97 | 6.53±0.74 | 7.53±0.06 | 13.33±0.58 | 25.72±3.88 | 9.00±1.00 | 1.07±0.12 | 7.00±1.41 | 0.49±0.1 | 1.85±0.09 | 1.07±0.05 | 1.66±0.18 | 0.43±0.03 | 3.83±0.11 |
| 11 | 25.73±0.55 | 29.53±0.74 | 6.37±0.12 | 3.63±0.25 | 14.67±5.51 | 48.73±12.29 | 14.35±1.91 | 1.03±0.25 | 9.00±1.73 | 0.96±0.25 | 2.69±0.08 | 1.08±0.06 | 1.85±0.09 | 0.48±0.04 | 3.84±0.06 |
| 12 | 25.30±0.87 | 28.93±1.19 | 6.43±1.25 | 6.53±2.05 | 11.67±2.52 | 44.08±0.30 | 14.50±0.71 | 0.60±0.14 | 5.00±0 | 0.33±0.06 | 1.02±0.16 | 1.29±0.04 | 1.90±0.26 | 0.53±0.06 | 3.58±0.16 |
| 13 | 22.33±1.7 | 22.13±4.14 | 4.60±0.80 | 5.23±1.14 | 14.00±5.66 | 43.85±6.69 | 12.00±0 | 0.70±0 | 6.00±0 | 0.48±0.04 | 1.46±0.15 | 1.08±0.04 | 1.76±0.20 | 0.47±0.05 | 3.77±0.04 |
| 14 | 25.70±0.61 | 25.37±1.65 | 4.17±0.40 | 2.57±0.57 | 17.00±11.31 | 60.54±16.95 | 9.60±0 | 2.10±0 | 14.00±0 | 1.39±0.06 | 4.50±0.50 | 1.18±0.04 | 1.86±0.11 | 0.49±0.05 | 3.79±0.08 |
| 15 | 24.90±1.71 | 22.53±1.17 | 4.50±0.36 | 5.87±1.68 | 13.33±2.31 | 51.22±5.15 | 6.77±1.97 | 0.77±0.12 | 4.33±1.53 | 0.60±0.05 | 2.10±0.29 | 1.45±0.16 | 1.76±0.12 | 0.45±0.06 | 3.87±0.09 |
| 16 | 26.23±0.38 | 23.23±1.81 | 4.90±0.61 | 3.67±0.76 | 17.33±5.51 | 46.14±4.44 | 6.00±1.41 | 2.05±0.49 | 9.00±0 | 1.07±0.16 | 3.36±0.37 | 1.08±0.03 | 1.73±0.09 | 0.45±0.06 | 3.81±0.07 |
| 17 | 24.40±4.06 | 30.57±3.30 | 6.50±0.62 | 12.90±1.65 | 16.33±4.16 | 33.36±6.33 | 16.67±1.04 | 1.10±0.10 | 8.00±1.00 | 0.89±0.13 | 2.47±0.16 | 1.03±0.09 | 1.76±0.13 | 0.43±0.03 | 4.06±0.08 |
| 18 | 23.53±2.06 | 26.13±1.62 | 4.87±0.29 | 3.43±0.75 | 15.33±3.51 | 47.24±8.00 | 19.67±4.73 | 0.67±0.29 | 8.00±2.00 | 0.99±0.13 | 2.88±0.41 | 1.20±0.09 | 1.88±0.08 | 0.45±0.05 | 4.18±0.07 |
| 19 | 25.20±0.82 | 21.67±0.50 | 4.87±1.21 | 7.13±0.85 | 12.00±2.00 | 43.59±7.98 | 13.00±1.41 | 0.70±0.28 | 4.50±0.71 | 1.15±0.10 | 2.09±0.20 | 1.17±0.03 | 1.82±0.06 | 0.40±0.01 | 4.53±0.04 |
| 20 | 21.93±2.76 | 26.40±2.21 | 5.23±0.40 | 5.53±2.71 | 15.00±2.65 | 40.24±2.67 | 7.50±0.71 | 0.60±0.14 | 7.00±0 | 0.68±0.15 | 2.31±0.06 | 1.35±0.01 | 1.77±0.16 | 0.48±0.08 | 3.69±0.12 |
| 21 | 29.57±4.97 | 24.47±0.95 | 4.40±0.35 | 4.53±0.59 | 14.00±3.46 | 47.54±4.05 | 13.50±1.41 | 0.90±0.14 | 5.00±1.41 | 1.02±0.17 | 2.76±0.15 | 0.92±0.06 | 1.75±0.14 | 0.42±0.05 | 4.17±0.10 |
| 22 | 21.93±1.55 | 22.47±2.05 | 5.93±0.57 | 5.73±0.68 | 14.67±4.04 | 71.05±6.46 | 11.00±2.12 | 1.15±0.07 | 8.00±2.83 | 1.04±0.17 | 3.59±0.23 | 1.12±0 | 1.78±0.14 | 0.53±0.06 | 3.38±0.10 |
| 23 | 24.97±3.40 | 23.90±0.52 | 6.10±0.46 | 3.40±0.53 | 15.00±3.00 | 48.61±9.55 | 3.93±0.40 | 1.03±0.15 | 8.00±1.00 | 0.86±0.08 | 3.55±0.38 | 1.04±0.06 | 1.80±0.09 | 0.46±0.04 | 3.92±0.06 |
| 24 | 26.23±2.50 | 30.93±3.33 | 5.57±0.46 | 8.00±0.53 | 13.33±3.79 | 54.32±5.64 | 9.65±0.49 | 1.15±0.07 | 9.00±1.41 | 0.99±0.16 | 3.02±0.16 | 1.05±0.13 | 1.78±0.10 | 0.48±0.06 | 3.68±0.02 |
| 25 | 29.20±3.54 | 27.77±2.14 | 4.47±0.40 | 5.50±0.28 | 14.67±6.43 | 70.30±5.57 | 15.65±0.92 | 1.10±0.14 | 7.50±0.71 | 0.68±0.07 | 2.34±0.23 | 1.21±0.10 | 1.85±0.28 | 0.48±0.11 | 3.82±0.18 |
| 26 | 27.10±3.82 | 29.10±0.53 | 5.67±0.81 | 6.10±1.23 | 15.67±1.15 | 46.79±5.78 | 17.60±2.97 | 0.90±0.28 | 9.50±2.12 | 0.48±0.04 | 1.73±0.03 | 1.04±0.11 | 1.64±0.06 | 0.47±0.07 | 3.53±0.06 |
| 27 | 20.03±3.06 | 24.57±1.35 | 5.87±0.15 | 6.97±0.72 | 16.00±6.08 | 60.91±9.37 | 9.75±1.77 | 1.20±0 | 10.00±1.41 | 1.05±0.10 | 3.75±0.31 | 1.17±0.09 | 1.57±0.05 | 0.53±0.04 | 2.95±0.05 |
| 28 | 14.03±0.95 | 23.13±2.73 | 4.77±0.40 | 4.77±0.38 | 9.67±1.53 | 47.2±21.72 | 6.60±0 | 1.50±0 | 6.00±0 | 0.92±0.10 | 3.54±0.04 | 0.49±0.01 | 1.02±0.05 | 0.42±0.04 | 2.42±0.04 |

^a^Seed Number

**Sequence of ITS2-2F(1~28)**

>*I. indigotica*（1）GAGTGACTCGAGTCTTTGACGCAAGTTGCGCCCTAAGCCTTCTGGCCGAGGGCACGTCTGCCTGGGTGTCACAAATCGTCGTCCCCCCATCCTCTCGAGGATAATGGACGGAAGCTGGTCTCCCGTGTGTTACCGCACGCGGTTGGCCAAAATCCGAGCTAAGGACGCAAGGAGCGTCTCGACATGCGGTGGTGAATTAAAACCTCGTCATACCGTTGGCCGCTCCTGTCCTGATGCTCTCGATGACCCAAAGTCCTCAACGCGACCCCAGGTCAGGCGGGATCACCCGCTGAGTTTAAGCATATCAATAAGCGGAGGAAAAGAAACTAACAAGGATTCCCTTAGTAACGGCGAGCGAACCGGGAAGAGCCCAGCTTGAAAATCGGACGTCTTCGGCGTTCGAA

>*I. indigotica*（2）

TAATGGTGACTCGAGTCTTTGACGCAAGTTGCGCCCTAAGCCTTCTGGCCGAGGGCACGTCTGCCTGGGTGTCACAAATCGTCGTCCCCCCATCCTCTCGAGGATAATGGACGGAAGCTGGTCTCCCGTGTGTTACCGCACGCGGTTGGCCAAAATCCGAGCTAAGGACGCAAGGAGCGTCTCGACATGCGGTGGTGAATTAAAACCTCGTCATACCGTTGGCCGCTCCTGTCCTGATGCTCTCGATGACCCAAAGTCCTCAACGCGACCCCAGGTCAGGCGGGATCACCCGCTGAGTTTAAGCATATCAATAAGCGGAGGAAAAGAAACTAACAAGGATTCCCTTAGTAACGGCGAGCGAACCGGGAAGAGCCCAGCTTGAAAATCGGACGTCTTCGGCGTTCGAATTGTAGTCTGAAGAAAGCGGTCAAAA

>*I. indigotica*（3）GAGTGGACTCGAGTCTTTGACGCAAGTTGCGCCCTAAGCCTTCTGGCCGAGGGCACGTCTGCCTGGGTGTCACAAATCGTCGTCCCCCCATCCTCTCGAGGATAATGGACGGAAGCTGGTCTCCCGTGTGTTACCGCACGCGGTTGGCCAAAATCCGAGCTAAGGACGCAAGGAGCGTCTCGACATGCGGTGGTGAATTAAAACCTCGTCATACCGTTGGCCGCTCCTGTCCTGATGCTCTCGATGACCCAAAGTCCTCAACGCGACCCCAGGTCAGGCGGGATCACCCGCTGAGTTTAAGCATATCAATAAGCGGAGGAAAAGAAACTAACAAGGATTCCCTTAGTAACGGCGAGCGAACCGGGAAGAGCCCAGCTTGAAAATCGGACGTCTTCGG

>*I. indigotica*（4）

CAGTGACTCGAGTCTTTGACGCAAGTTGCGCCCTAAGCCTTCTGGCCGAGGGCACGTCTGCCTGGGTGTCACAAATCGTCGTCCCCCCATCCTCTCGAGGATAATGGACGGAAGCTGGTCTCCCGTGTGTTACCGCACGCGGTTGGCCAAAATCCGAGCTAAGGACGCAAGGAGCGTCTCGACATGCGGTGGTGAATTAAAACCTCGTCATACCGTTGGCCGCTCCTGTCCTGATGCTCTCGATGACCCAAAGTCCTCAACGCGACCCCAGGTCAGGCGGGATCACCCGCTGAGTTTAAGCATATCAATAAGCGGAGGAAAAGAAACTAACAAGGATTCCCTTAGTAACGGCGAGCGAACCGGGAAGAGCCCAGCTTGAAAATCGGACGTCTTCG

>*I. indigotica*（5）CACTGACTCGAGTCTTTGACGCAAGTTGCGCCCTAAGCCTTCTGGCCGAGGGCACGTCTGCCTGGGTGTCACAAATCGTCGTCCCCCCATCCTCTCGAGGATAATGGACGGAAGCTGGTCTCCCGTGTGTTACCGCACGCGGTTGGCCAAAATCCGAGCTAAGGACGCAAGGAGCGTCTCGACATGCGGTGGTGAATTAAAACCTCGTCATACCGTTGGCCGCTCCTGTCCTGATGCTCTCGATGACCCAAAGTCCTCAACGCGACCCCAGGTCAGGCGGGATCACCCGCTGAGTTTAAGCATATCAATAAGCGGAGGAAAAGAAACTAACAAGGATTCCCTTAGTAACGGCGAGCGAACCGGGAAGAGCCCAGCTTGAAAATCGGACGTCTTC

>*I. indigotica*（6）

CAAGTGACTCGAGTCTTTGACGCAAGTTGCGCCCTAAGCCTTCTGGCCGAGGGCACGTCTGCCTGGGTGTCACAAATCGTCGTCCCCCCATCCTCTCGAGGATAATGGACGGAAGCTGGTCTCCCGTGTGTTACCGCACGCGGTTGGCCAAAATCCGAGCTAAGGACGCAAGGAGCGTCTCGACATGCGGTGGTGAATTAAAACCTCGTCATACCGTTGGCCGCTCCTGTCCTGATGCTCTCGATGACCCAAAGTCCTCAACGCGACCCCAGGTCAGGCGGGATCACCCGCTGAGTTTAAGCATATCAATAAGCGGAGGAAAAGAAACTAACAAGGATTCCCTTAGTAACGGCGAGCGAACCGGGAAGAGCCCAGCTTGAAAATCGGACGTCTTCGGCG

>*I. indigotica*（7）CGATTGGTGACTCGAGTCTTTGACGCAAGTTGCGCCCTAAGCCTTCTGGCCGAGGGCACGTCTGCCTGGGTGTCACAAATCGTCGTCCCCCCATCCTCTCGAGGATAATGGACGGAAGCTGGTCTCCCGTGTGTTACCGCACGCGGTTGGCCAAAATCCGAGCTAAGGACGCAAGGAGCGTCTCGACATGCGGTGGTGAATTAAAACCTCGTCATACCGTTGGCCGCTCCTGTCCTGATGCTCTCGATGACCCAAAGTCCTCAACGCGACCCCAGGTCAGGCGGGATCACCCGCTGAGTTTAAGCATATCAATAAGCGGAGGAAAAGAAACTAACAAGGATTCCCTTAGTAACGGCGAGCGAACCGGGAAGAGCCCAGCTTGAAAATCGGACGTCTTCGGCGTTCGAATTGTAGTCTGAAAAAAGCGGCCAAGC

>*I. indigotica*（8）GTGTGGGACTCGAGTCTTTGACGCAGTTGCGCCCTAAGCCTTCTGGCCGAGGGCACGTCTGCCTGGGTGTCACAAATCGTCGTCCCCCCATCCTCTCGAGGATAATGGACGGAAGCTGGTCTCCCGTGTGTTACCGCACGCGGTTGGCCAAAATCCGAGCTAAGGACGCAAGGAGCGTCTCGACATGCGGTGGTGAATTAAAACCTCGTCATACCGTTGGCCGCTCCTGTCCTGATGCTCTCGATGACCCAAAGTCCTCAACGCGACCCCAGGTCAGGCGGGATCACCCGCTGAGTTTAAGCATATCAATAAGCGGAGGAAAAGAAACTAACAAGGATTCCCTTAGTAACGGCGAGCGAACCGGGAAGAGCCCAGCTTGAAAATCGGACGTCTTCGGCGT

>*I. indigotica*（9）GTATGGGACTCGAGTCTTTGACGCAAGTTGCGCCCTAAGCCTTCTGGCCGAGGGCACGTCTGCCTGGGTGTCACAAATCGTCGTCCCCCCATCCTCTCGAGGATAATGGACGGAAGCTGGTCTCCCGTGTGTTACCGCACGCGGTTGGCCAAAATCCGAGCTAAGGACGCAAGGAGCGTCTCGACATGCGGTGGTGAATTAAAACCTCGTCATACCGTTGGCCGCTCCTGTCCTGATGCTCTCGATGACCCAAAGTCCTCAACGCGACCCCAGGTCAGGCGGGATCACCCGCTGAGTTTAAGCATATCAATAAGCGGAGGAAAAGAAACTAACAAGGATTCCCTTAGTAACGGCGAGCGAACCGGGAAGAGCCCAGCTTGAAAATCGGACGTCTTCGGCGT

>*I. indigotica*（10）AGACGGACTCGAGTCTTTGACGCAAGTTGCGCCCTAAGCCTTCTGGCCGAGGGCACGTCTGCCTGGGTGTCACAAATCGTCGTCCCCCCATCCTCTCGAGGATAATGGACGGAAGCTGGTCTCCCGTGTGTTACCGCACGCGGTTGGCCAAAATCCGAGCTAAGGACGCAAGGAGCGTCTCGACATGCGGTGGTGAATTAAAACCTCGTCATACCGTTGGCCGCTCCTGTCCTGATGCTCTCGATGACCCAAAGTCCTCAACGCGACCCCAGGTCAGGCGGGATCACCCGCTGAGTTTAAGCATATCAATAAGCGGAGGAAAAGAAACTAACAAGGATTCCCTTAGTAACGGCGAGCGAACCGGGAAGAGCCCAGCTTGAAAATCGGACGTCTTCGGCG

>*I. indigotica*（11）GGATGGGACTCGAGTCTTTGACGCAAGTTGCGCCCTAAGCCTTCTGGCCGAGGGCACGTCTGCCTGGGTGTCACAAATCGTCGTCCCCCCATCCTCTCGAGGATAATGGACGGAAGCTGGTCTCCCGTGTGTTACCGCACGCGGTTGGCCAAAATCCGAGCTAAGGACGCAAGGAGCGTCTCGACATGCGGTGGTGAATTAAAACCTCGTCATACCGTTGGCCGCTCCTGTCCTGATGCTCTCGATGACCCAAAGTCCTCAACGCGACCCCAGGTCAGGCGGGATCACCCGCTGAGTTTAAGCATATCAATAAGCGGAGGAAAAGAAACTAACAAGGATTCCCTTAGTAACGGCGAGCGAACCGGGAAGAGCCCAGCTTGAAAATCGGACGTCTTCGGCGTTCG

>*I. indigotica*（12） AACTGACTCGAGTCTTTGACGCAAGTTGCGCCCTAAGCCTTCTGGCCGAGGGCACGTCTGCCTGGGTGTCACAAATCGTCGTCCCCCCATCCTCTCGAGGATAATGGACGGAAGCTGGTCTCCCGTGTGTTACCGCACGCGGTTGGCCAAAATCCGAGCTAAGGACGCAAGGAGCGTCTCGACATGCGGTGGTGAATTAAAACCTCGTCATACCGTTGGCCGCTCCTGTCCTGATGCTCTCGATGACCCAAAGTCCTCAACGCGACCCCAGGTCAGGCGGGATCACCCGCTGAGTTTAAGCATATCAATAAGCGGAGGAAAAGAAACTAACAAGGATTCCCTTAGTAACGGCGAGCGAACCGGGAAGAGCCCAGCTTGAAAATCGGACGTCTTCGGC

>*I. indigotica*（13）

ATACGGACTCGAGTCTTTGACGCAAGTTGCGCCCTAAGCCTTCTGGCCGAGGGCACGTCTGCCTGGGTGTCACAAATCGTCGTCCCCCCATCCTCTCGAGGATAATGGACGGAAGCTGGTCTCCCGTGTGTTACCGCACGCGGTTGGCCAAAATCCGAGCTAAGGACGCAAGGAGCGTCTCGACATGCGGTGGTGAATTAAAACCTCGTCATACCGTTGGCCGCTCCTGTCCTGATGCTCTCGATGACCCAAAGTCCTCAACGCGACCCCAGGTCAGGCGGGATCACCCGCTGAGTTTAAGCATATCAATAAGCGGAGGAAAAGAAACTAACAAGGATTCCCTTAGTAACGGCGAGCGAACCGGGAAGAGCCCAGCTTGAAAATCGGACGTCTTCGG

>*I. indigotica*（14）

AACGGACTCGAGTCTTTGACGCAAGTTGCGCCCTAAGCCTTCTGGCCGAGGGCACGTCTGCCTGGGTGTCACAAATCGTCGTCCCCCCATCCTCTCGAGGATAATGGACGGAAGCTGGTCTCCCGTGTGTTACCGCACGCGGTTGGCCAAAATCCGAGCTAAGGACGCAAGGAGCGTCTCGACATGCGGTGGTGAATTAAAACCTCGTCATACCGTTGGCCGCTCCTGTCCTGATGCTCTCGATGACCCAAAGTCCTCAACGCGACCCCAGGTCAGGCGGGATCACCCGCTGAGTTTAAGCATATCAATAAGCGGAGGAAAAGAAACTAACAAGGATTCCCTTAGTAACGGCGAGCGAACCGGGAAGAGCCCAGCTTGAAAATCGGACGTCTTCGG

>*I. indigotica*（15）GGGCATTGTGGGACTCGAGTCTTTGACGCAAGTTGCGCCCTAAGCCTTCTGGCCGAGGGCACGTCTGCCTGGGTGTCACAAATCGTCGTCCCCCCATCCTCTCGAGGATAATGGACGGAAGCTGGTCTCCCGTGTGTTACCGCACGCGGTTGGCCAAAATCCGAGCTAAGGACGCAAGGAGCGTCTCGACATGCGGTGGTGAATTAAAACCTCGTCATACCGTTGGCCGCTCCTGTCCTGATGCTCTCGATGACCCAAAGTCCTCAACGCGACCCCAGGTCAGGCGGGATCACCCGCTGAGTTTAAGCATATCAATAAGCGGAGGAAAAGAAACTAACAAGGATTCCCTTAGTAACGGCGAGCGAACCGGGAAGAGCCCAGCTTGAAAATCGGACGTCTTCGGCGTTCGAATTGTAGTCTGAAGAAGCGTCAAA

>*I. indigotica*（16）GGACTGATGGGACTCGAGTCTTTGACGCAAGTTGCGCCCTAAGCCTTCTGGCCGAGGGCACGTCTGCCTGGGTGTCACAAATCGTCGTCCCCCCATCCTCTCGAGGATAATGGACGGAAGCTGGTCTCCCGTGTGTTACCGCACGCGGTTGGCCAAAATCCGAGCTAAGGACGCAAGGAGCGTCTCGACATGCGGTGGTGAATTAAAACCTCGTCATACCGTTGGCCGCTCCTGTCCTGATGCTCTCGATGACCCAAAGTCCTCAACGCGACCCCAGGTCAGGCGGGATCACCCGCTGAGTTTAAGCATATCAATAAGCGGAGGAAAAGAAACTAACAAGGATTCCCTTAGTAACGGCGAGCGAACCGGGAAGAGCCCAGCTTGAAAATCGGACGTCTTCGGCGTTCGAATTGTAGTCTGAAGAAGCGTCA

>*I. indigotica*（17）ACGATGTGACTCGAGTCTTTGACGCAAGTTGCGCCCTAAGCCTTCTGGCCGAGGGCACGTCTGCCTGGGTGTCACAAATCGTCGTCCCCCCATCCTCTCGAGGATAATGGACGGAAGCTGGTCTCCCGTGTGTTACCGCACGCGGTTGGCCAAAATCCGAGCTAAGGACGCAAGGAGCGTCTCGACATGCGGTGGTGAATTAAAACCTCGTCATACCGTTGGCCGCTCCTGTCCTGATGCTCTCGATGACCCAAAGTCCTCAACGCGACCCCAGGTCAGGCGGGATCACCCGCTGAGTTTAAGCATATCAATAAGCGGAGGAAAAGAAACTAACAAGGATTCCCTTAGTAACGGCGAGCGAACCGGGAAGAGCCCAGCTTGAAAATCGGACGTCTTCGGCGTTCGAATTGTAGTCTGAAGAAAGCGTCAAA

>*I. indigotica*（18）TCTAGGCCCGCTGCGTGGACTCGAGTCTTTGACGCAAGTTGCGCCCTAAGCCTTCTGGCCGAGGGCACGTCTGCCTGGGTGTCACAAATCGTCGTCCCCCCATCCTCTCGAGGATAATGGACGGAAGCTGGTCTCCCGTGTGTTACCGCACGCGGTTGGCCAAAATCCGAGCTAAGGACGCAAGGAGCGTCTCGACATGCGGTGGTGAATTAAAACCTCGTCATACCGTTGGCCGCTCCTGTCCTGATGCTCTCGATGACCCAAAGTCCTCAACGCGACCCCAGGTCAGGCGGGATCACCCGCTGAGTTTAAGCATATCAATAAGCGGAGGAAAAGAAACTAACAAGGATTCCCTTAGTAACGGCGAGCGAACCGGGAAGAGCCCAGCTTGAAAATCGGACGTCTTCGGCGTTCGAATTGTAGTCTGGAAGAAGCGTCA

>*I. indigotica*（19）TGCCCCCCCCCGTGTGACTCGAGTCTTTGACGCAGTTGCGCCCTAAGCCTTCTGGCCGAGGGCACGTCTGCCTGGGTGTCACAAATCGTCGTCCCCCCATCCTCTCGAGGATAATGGACGGAAGCTGGTCTCCCGTGTGTTACCGCACGCGGTTGGCCAAAATCCGAGCTAAGGACGCAAGGAGCGTCTCGACATGCGGTGGTGAATTAAAACCTCGTCATACCGTTGGCCGCTCCTGTCCTGATGCTCTCGATGACCCAAAGTCCTCAACGCGACCCCAGGTCAGGCGGGATCACCCGCTGAGTTTAAGCATATCAATAAGCGGAGGAAAAGAAACTAACAAGGATTCCCTTAGTAACGGCGAGCGAACCGGGAAGAGCCCAGCTTGAAAATCGGACGTCTTCGGCGTTCGAATTGTAGTCTGGAGAAGCGTCAAG

>*I. indigotica*（20）

CATCAACCTGCGGCGCGTAATCGAGTCTTTGACGCAGTTGCGCCCTAAGCCTTCTGGCCGAGGGCACGTCTGCCTGGGTGTCACAAATCGTCGTCCCCCCATCCTCTCGAGGATAATGGACGGAAGCTGGTCTCCCGTGTGTTACCGCACGCGGTTGGCCAAAATCCGAGCTAAGGACGCAAGGAGCGTCTCGACATGCGGTGGTGAATTAAAACCTCGTCATACCGTTGGCCGCTCCTGTCCTGATGCTCTCGATGACCCAAAGTCCTCAACGCGACCCCAGGTCAGGCGGGATCACCCGCTGAGTTTAAGCATATCAATAAGCGGAGGAAAAGAAACTAACAAGGATTCCCTTAGTAACGGCGAGCGAACCGGGAAGAGCCCAGCTTGAAAATCGGACGTCTTCGGCGTTCGAATTGTAGTCTGGAGAAGCGTCAAA

>*I. indigotica*（21）

CGAAGGACACACTTGTGTACTCGAGTCTTTGACGCAGTTGCGCCCTAAGCCTTCTGGCCGAGGGCACGTCTGCCTGGGTGTCACAAATCGTCGTCCCCCCATCCTCTCGAGGATAATGGACGGAAGCTGGTCTCCCGTGTGTTACCGCACGCGGTTGGCCAAAATCCGAGCTAAGGACGCAAGGAGCGTCTCGACATGCGGTGGTGAATTAAAACCTCGTCATACCGTTGGCCGCTCCTGTCCTGATGCTCTCGATGACCCAAAGTCCTCAACGCGACCCCAGGTCAGGCGGGATCACCCGCTGAGTTTAAGCATATCAATAAGCGGAGGAAAAGAAACTAACAAGGATTCCCTTAGTAACGGCGAGCGAACCGGGAAGAGCCCAGCTTGAAAATCGGACGTCTTCGGCGTTCGAATTGTAGTCTGGAGAAGCGTCAAA

>*I. indigotica*（22）

TAAACGGGCGCCGCTGTGGGAATCGAGTCTTTGACGCAAGTTGCGCCCTAAGCCTTCTGGCCGAGGGCACGTCTGCCTGGGTGTCACAAATCGTCGTCCCCCCATCCTCTCGAGGATAATGGACGGAAGCTGGTCTCCCGTGTGTTACCGCACGCGGTTGGCCAAAATCCGAGCTAAGGACGCAAGGAGCGTCTCGACATGCGGTGGTGAATTAAAACCTCGTCATACCGTTGGCCGCTCCTGTCCTGATGCTCTCGATGACCCAAAGTCCTCAACGCGACCCCAGGTCAGGCGGGATCACCCGCTGAGTTTAAGCATATCAATAAGCGGAGGAAAAGAAACTAACAAGGATTCCCTTAGTAACGGCGAGCGAACCGGGAAGAGCCCAGCTTGAAAATCGGACGTCTTCGGCGTTCGAATTGTAGTCTGGAGAAGCGTCAAC

>*I. indigotica*（23）

TGACTGACTCGAGTCTTTGACGCAGTTGCGCCCTAAGCCTTCTGGCCGAGGGCACGTCTGCCTGGGTGTCACAAATCGTCGTCCCCCCATCCTCTCGAGGATAATGGACGGAAGCTGGTCTCCCGTGTGTTACCGCACGCGGTTGGCCAAAATCCGAGCTAAGGACGCAAGGAGCGTCTCGACATGCGGTGGTGAATTAAAACCTCGTCATACCGTTGGCCGCTCCTGTCCTGATGCTCTCGATGACCCAAAGTCCTCAACGCGACCCCAGGTCAGGCGGGATCACCCGCTGAGTTTAAGCATATCAATAAGCGGAGGAAAAGAAACTAACAAGGATTCCCTTAGTAACGGCGAGCGAACCGGGAAGAGCCCAGCTTGAAAATCGGACGTCTTCGGCGTTCGAATTGTAGTCTGAAGAAAGCGTCAAA

>*I. indigotica*（24）

ATTGACTCGAGTCTTTGACGCAAGTTGCGCCCTAAGCCTTCTGGCCGAGGGCACGTCTGCCTGGGTGTCACAAATCGTCGTCCCCCCATCCTCTCGAGGATAATGGACGGAAGCTGGTCTCCCGTGTGTTACCGCACGCGGTTGGCCAAAATCCGAGCTAAGGACGCAAGGAGCGTCTCGACATGCGGTGGTGAATTAAAACCTCGTCATACCGTTGGCCGCTCCTGTCCTGATGCTCTCGATGACCCAAAGTCCTCAACGCGACCCCAGGTCAGGCGGGATCACCCGCTGAGTTTAAGCATATCAATAAGCGGAGGAAAAGAAACTAACAAGGATTCCCTTAGTAACGGCGAGCGAACCGGGAAGAGCCCAGCTTGAAAATCGGACGTCTTCGGCGTTCGAATTGTAGTCTGGAAA

>*I. indigotica*（25）

AATTGACTCGAGTCTTTGACGCAAGTTGCGCCCTAAGCCTTCTGGCCGAGGGCACGTCTGCCTGGGTGTCACAAATCGTCGTCCCCCCATCCTCTCGAGGATAATGGACGGAAGCTGGTCTCCCGTGTGTTACCGCACGCGGTTGGCCAAAATCCGAGCTAAGGACGCAAGGAGCGTCTCGACATGCGGTGGTGAATTAAAACCTCGTCATACCGTTGGCCGCTCCTGTCCTGATGCTCTCGATGACCCAAAGTCCTCAACGCGACCCCAGGTCAGGCGGGATCACCCGCTGAGTTTAAGCATATCAATAAGCGGAGGAAAAGAAACTAACAAGGATTCCCTTAGTAACGGCGAGCGAACCGGGAAGAGCCCAGCTTGAAAATCGGACGTCTTCGGCGTTCGAATTGTAGTCTGAAGAAAGCGTCAAA

>*I. indigotica*（26）

CCCCGACCTCGAGTCTTTGACGCAGTTGCGCCCTAAGCCTTCTGGCCGAGGGCACGTCTGCCTGGGTGTCACAAATCGTCGTCCCCCCATCCTCTCGAGGATAATGGACGGAAGCTGGTCTCCCGTGTGTTACCGCACGCGGTTGGCCAAAATCCGAGCTAAGGACGCAAGGAGCGTCTCGACATGCGGTGGTGAATTAAAACCTCGTCATACCGTTGGCCGCTCCTGTCCTGATGCTCTCGATGACCCAAAGTCCTCAACGCGACCCCAGGTCAGGCGGGATCACCCGCTGAGTTTAAGCATATCAATAAGCGGAGGAAAAGAAACTAACAAGGATTCCCTTAGTAACGGCGAGCGAACCGGGAAGAGCCCAGCTTGAAAATCGGACGTCTTCGGCGTTCGAATTGTAGTCTGAAAAA

>*I. indigotica*（27）

TTTCTATTGACTCGAGTCTTTGACGCAAGTTGCGCCCTAAGCCTTCTGGCCGAGGGCACGTCTGCCTGGGTGTCACAAATCGTCGTCCCCCCATCCTCTCGAGGATAATGGACGGAAGCTGGTCTCCCGTGTGTTACCGCACGCGGTTGGCCAAAATCCGAGCTAAGGACGCAAGGAGCGTCTCGACATGCGGTGGTGAATTAAAACCTCGTCATACCGTTGGCCGCTCCTGTCCTGATGCTCTCGATGACCCAAAGTCCTCAACGCGACCCCAGGTCAGGCGGGATCACCCGCTGAGTTTAAGCATATCAATAAGCGGAGGAAAAGAAACTAACAAGGATTCCCTTAGTAACGGCGAGCGAACCGGGAAGAGCCCAGCTTGAAAATCGGACGTCTTCGGCGTTCGAATTGTAGTCTGGAGAAGCGTCAAAC

>*I. tinctoria*（28）AGATCGTGACTCGAGTCTTTGACGCAAGTTGCGCCCTAAGCCTTCTGGCCGAGGGCACGTCTGCCTGGGTGTCACAAATCGTCGTCCCCCCATCCTCTCGAGGATAATGGACGGAAGCTGGTCTCCCGTGTGTTACCGCACGCGGTTGGCCAAAATCCGAGCTAAGGACGCAAGGAGCGTCCCGACATGCGGTGGTGAATTAAAACCTCGTCATACCGTCGGCCGCTCCTGTCCTGATGCTCTCGATGACCCAATGTCCTCAACGCGACCCCAGGTCAGGCGGGATCACCCGCTGAGTTTAAGCATATCAATAAGCGGAGGAAAAGAAACTAACAAGGATTCCCTTAGTAACGGCGAGCGAACCGGGAAGAGCCCAGCTTGAAAATCGGACGTCTTCGGCGTTCGAATTGTAGTCTGAAAAAAAGCGTCA

**Sequence of ITS2-p3(1~28)**

>*I. indigotica*（1）

GGACGAAGAGACGTAGCGATGCGATACTTGGTGTGAATTGCAGAATCCCGTGAACCATCGAGTCTTTGAACGCAAGTTGCGCCCTAAGCCTTCTGGCCGAGGGCACGTCTGCCTGGGTGTCACAAATCGTCGTCCCCCCATCCTCTCGAGGATAATGGACGGAAGCTGGTCTCCCGTGTGTTACCGCACGCGGTTGGCCAAAATCCGAGCTAAGGACGCAAGGAGCGTCTCGACATGCGGTGGTGAATTAAAACCTCGTCATACCGTTGGCCGCTCCTGTCCTGATGCTCTCGATGACCCAAAGTCCTCAACGCGACCCCAGGTCAGGCGGGATCACCCGCTGAGTTTAAGCATATCAATAAGC

>*I. indigotica*（2）

GGCTTATCGATGAGACGTAGCGAATGCGATACTTGGTGTGAATTGCAGAATCCCGTGAACCATCGAGTCTTTGAACGCAAGTTGCGCCCTAAGCCTTCTGGCCGAGGGCACGTCTGCCTGGGTGTCACAAATCGTCGTCCCCCCATCCTCTCGAGGATAATGGACGGAAGCTGGTCTCCCGTGTGTTACCGCACGCGGTTGGCCAAAATCCGAGCTAAGGACGCAAGGAGCGTCTCGACATGCGGTGGTGAATTAAAACCTCGTCATACCGTTGGCCGCTCCTGTCCTGATGCTCTCGATGACCCAAAGTCCTCAACGCGACCCCAGGTCAGGCGGGATCACCCGCTGAGTTTAAGCATATCAATAAGCGGAGAAAAAGAAACCAAAAAA

>*I. indigotica*（3）

CAGGCTACGATGAGACGTAGCGAATGCGATACTTGGTGTGATTGCAGAATCCCGTGAACCATCGAGTCTTTGAACGCAAGTTGCGCCCTAAGCCTTCTGGCCGAGGGCACGTCTGCCTGGGTGTCACAAATCGTCGTCCCCCCATCCTCTCGAGGATAATGGACGGAAGCTGGTCTCCCGTGTGTTACCGCACGCGGTTGGCCAAAATCCGAGCTAAGGACGCAAGGAGCGTCTCGACATGCGGTGGTGAATTAAAACCTCGTCATACCGTTGGCCGCTCCTGTCCTGATGCTCTCGATGACCCAAAGTCCTCAACGCGACCCCAGGTCAGGCGGGATCACCCGCTGAGTTTAAGCATATCAATAAG

>*I. indigotica*（4）

AGGGAGGACGAGAGACGTAGCGATGCGATACTTGGTGTGAATTGCAGAATCCCGTGAACCATCGAGTCTTTGAACGCAAGTTGCGCCCTAAGCCTTCTGGCCGAGGGCACGTCTGCCTGGGTGTCACAAATCGTCGTCCCCCCATCCTCTCGAGGATAATGGACGGAAGCTGGTCTCCCGTGTGTTACCGCACGCGGTTGGCCAAAATCCGAGCTAAGGACGCAAGGAGCGTCTCGACATGCGGTGGTGAATTAAAACCTCGTCATACCGTTGGCCGCTCCTGTCCTGATGCTCTCGATGACCCAAAGTCCTCAACGCGACCCCAGGTCAGGCGGGATCACCCGCTGAGTTTAAGCATATCAA

>*I. indigotica*（5）

AGGGAGGACGAGAGACGTAGCGATGCGATACTTGGTGTGAATTGCAGAATCCCGTGAACCATCGAGTCTTTGAACGCAAGTTGCGCCCTAAGCCTTCTGGCCGAGGGCACGTCTGCCTGGGTGTCACAAATCGTCGTCCCCCCATCCTCTCGAGGATAATGGACGGAAGCTGGTCTCCCGTGTGTTACCGCACGCGGTTGGCCAAAATCCGAGCTAAGGACGCAAGGAGCGTCTCGACATGCGGTGGTGAATTAAAACCTCGTCATACCGTTGGCCGCTCCTGTCCTGATGCTCTCGATGACCCAAAGTCCTCAACGCGACCCCAGGTCAGGCGGGATCACCCGCTGAGTTTAAGCATATCAA

>*I. indigotica*（6）

GGGGGACGAGAGACGTAGCGAATGCGATACTTGGTGTGATTGCAGAATCCCGTGAACCATCGAGTCTTTGAACGCAAGTTGCGCCCTAAGCCTTCTGGCCGAGGGCACGTCTGCCTGGGTGTCACAAATCGTCGTCCCCCCATCCTCTCGAGGATAATGGACGGAAGCTGGTCTCCCGTGTGTTACCGCACGCGGTTGGCCAAAATCCGAGCTAAGGACGCAAGGAGCGTCTCGACATGCGGTGGTGAATTAAAACCTCGTCATACCGTTGGCCGCTCCTGTCCTGATGCTCTCGATGACCCAAAGTCCTCAACGCGACCCCAGGTCAGGCGGGATCACCCGCTGAGTTTAAGCAT

>*I. indigotica*（7）

GGGCTACGAGAGACGTAGCGATGCGATACTTGGTGTGATTGCAGAATCCCGTGAACCATCGAGTCTTTGAACGCAAGTTGCGCCCTAAGCCTTCTGGCCGAGGGCACGTCTGCCTGGGTGTCACAAATCGTCGTCCCCCCATCCTCTCGAGGATAATGGACGGAAGCTGGTCTCCCGTGTGTTACCGCACGCGGTTGGCCAAAATCCGAGCTAAGGACGCAAGGAGCGTCTCGACATGCGGTGGTGAATTAAAACCTCGTCATACCGTTGGCCGCTCCTGTCCTGATGCTCTCGATGACCCAAAGTCCTCAACGCGACCCCAGGTCAGGCGGGATCACCCGCTGAGTTTAAG

>*I. indigotica*（8）

CGGGGGCAGATGAGACGTAGCGAATGCGATACTTGGTGTGAATTGCAGAATCCCGTGAACCATCGAGTCTTTGAACGCAAGTTGCGCCCTAAGCCTTCTGGCCGAGGGCACGTCTGCCTGGGTGTCACAAATCGTCGTCCCCCCATCCTCTCGAGGATAATGGACGGAAGCTGGTCTCCCGTGTGTTACCGCACGCGGTTGGCCAAAATCCGAGCTAAGGACGCAAGGAGCGTCTCGACATGCGGTGGTGAATTAAAACCTCGTCATACCGTTGGCCGCTCCTGTCCTGATGCTCTCGATGACCCAAAGTCCTCAACGCGACCCCAGGTCAGGCGGGATCACCCGCTGAGTTTAAGCATATC

>*I. indigotica*（9）

CCAGCGATGAGACGTAGCGATGCGATACTTGGTGTGAATTGCAGAATCCCGTGAACCATCGAGTCTTTGAACGCAAGTTGCGCCCTAAGCCTTCTGGCCGAGGGCACGTCTGCCTGGGTGTCACAAATCGTCGTCCCCCCATCCTCTCGAGGATAATGGACGGAAGCTGGTCTCCCGTGTGTTACCGCACGCGGTTGGCCAAAATCCGAGCTAAGGACGCAAGGAGCGTCTCGACATGCGGTGGTGAATTAAAACCTCGTCATACCGTTGGCCGCTCCTGTCCTGATGCTCTCGATGACCCAAAGTCCTCAACGCGACCCCAGGTCAGGCGGGATCACCCGCTGAGTTTAAGCATATCAA

>*I. indigotica*（10）

AGGGGCACGATGAGACGTAGCGATGCGATACTTGGTGTGAATTGCAGAATCCCGTGAACCATCGAGTCTTTGAACGCAAGTTGCGCCCTAAGCCTTCTGGCCGAGGGCACGTCTGCCTGGGTGTCACAAATCGTCGTCCCCCCATCCTCTCGAGGATAATGGACGGAAGCTGGTCTCCCGTGTGTTACCGCACGCGGTTGGCCAAAATCCGAGCTAAGGACGCAAGGAGCGTCTCGACATGCGGTGGTGAATTAAAACCTCGTCATACCGTTGGCCGCTCCTGTCCTGATGCTCTCGATGACCCAAAGTCCTCAACGCGACCCCAGGTCAGGCGGGATCACCCGCTGAGTTTAAGCATATC

>*I. indigotica*（11）

CGGGGGGACGATGAGACGTAGCGATGCGATACTTGGTGTGAATTGCAGAATCCCGTGAACCATCGAGTCTTTGAACGCAAGTTGCGCCCTAAGCCTTCTGGCCGAGGGCACGTCTGCCTGGGTGTCACAAATCGTCGTCCCCCCATCCTCTCGAGGATAATGGACGGAAGCTGGTCTCCCGTGTGTTACCGCACGCGGTTGGCCAAAATCCGAGCTAAGGACGCAAGGAGCGTCTCGACATGCGGTGGTGAATTAAAACCTCGTCATACCGTTGGCCGCTCCTGTCCTGATGCTCTCGATGACCCAAAGTCCTCAACGCGACCCCAGGTCAGGCGGGATCACCCGCTGAGTTTAAGCATATCA

>*I. indigotica*（12）

GGGGGGAGATGAGACGTAGCGAATGCGATACTTGGTGTGAATTGCAGAATCCCGTGAACCATCGAGTCTTTGAACGCAAGTTGCGCCCTAAGCCTTCTGGCCGAGGGCACGTCTGCCTGGGTGTCACAAATCGTCGTCCCCCCATCCTCTCGAGGATAATGGACGGAAGCTGGTCTCCCGTGTGTTACCGCACGCGGTTGGCCAAAATCCGAGCTAAGGACGCAAGGAGCGTCTCGACATGCGGTGGTGAATTAAAACCTCGTCATACCGTTGGCCGCTCCTGTCCTGATGCTCTCGATGACCCAAAGTCCTCAACGCGACCCCAGGTCAGGCGGGATCACCCGCTGAGTTTAAGCATATCAATAAGC

>*I. indigotica*（13）

GGGGGGAAGATGAGACGTAGCGAATGCGATACTTGGTGTGAATTGCAGAATCCCGTGAACCATCGAGTCTTTGAACGCAAGTTGCGCCCTAAGCCTTCTGGCCGAGGGCACGTCTGCCTGGGTGTCACAAATCGTCGTCCCCCCATCCTCTCGAGGATAATGGACGGAAGCTGGTCTCCCGTGTGTTACCGCACGCGGTTGGCCAAAATCCGAGCTAAGGACGCAAGGAGCGTCTCGACATGCGGTGGTGAATTAAAACCTCGTCATACCGTTGGCCGCTCCTGTCCTGATGCTCTCGATGACCCAAAGTCCTCAACGCGACCCCAGGTCAGGCGGGATCACCCGCTGAGTTTAAGCATATC

>*I. indigotica*（14）

GAGGGCCACGATGAGACGTAGCGATGCGATACTTGGTGTGAATTGCAGAATCCCGTGAACCATCGAGTCTTTGAACGCAA

GTTGCGCCCTAAGCCTTCTGGCCGAGGGCACGTCTGCCTGGGTGTCACAAATCGTCGTCCCCCCATCCTCTCGAGGATAA

TGGACGGAAGCTGGTCTCCCGTGTGTTACCGCACGCGGTTGGCCAAAATCCGAGCTAAGGACGCAAGGAGCGTCTCGACA

TGCGGTGGTGAATTAAAACCTCGTCATACCGTTGGCCGCTCCTGTCCTGATGCTCTCGATGACCCAAAGTCCTCAACGCG

ACCCCAGGTCAGGCGGGATCACCCGCTGAGTTTAAGCATATCAATAAGCGGAGAA

>*I. indigotica*（15）

GCCTTGATCGATGAGACGTAGCGATGCGATACTTGGTGTGAATTGCAGAATCCCGTGAACCATCGAGTCTTTGAACGCAAGTTGCGCCCTAAGCCTTCTGGCCGAGGGCACGTCTGCCTGGGTGTCACAAATCGTCGTCCCCCCATCCTCTCGAGGATAATGGACGGAAGCTGGTCTCCCGTGTGTTACCGCACGCGGTTGGCCAAAATCCGAGCTAAGGACGCAAGGAGCGTCTCGACATGCGGTGGTGAATTAAAACCTCGTCATACCGTTGGCCGCTCCTGTCCTGATGCTCTCGATGACCCAAAGTCCTCAACGCGACCCCAGGTCAGGCGGGATCACCCGCTGAGTTTAAGCATATCAATAAGCGGAGGAAAAAGAAACTAAAAGG

>*I. indigotica*（16）

CCGGCGTAATCGATGAGACGTAGCGAATGCGATACTTGGTGTGAATTGCAGAATCCCGTGAACCATCGAGTCTTTGAACGCAAGTTGCGCCCTAAGCCTTCTGGCCGAGGGCACGTCTGCCTGGGTGTCACAAATCGTCGTCCCCCCATCCTCTCGAGGATAATGGACGGAAGCTGGTCTCCCGTGTGTTACCGCACGCGGTTGGCCAAAATCCGAGCTAAGGACGCAAGGAGCGTCTCGACATGCGGTGGTGAATTAAAACCTCGTCATACCGTTGGCCGCTCCTGTCCTGATGCTCTCGATGACCCAAAGTCCTCAACGCGACCCCAGGTCAGGCGGGATCACCCGCTGAGTTTAAGCATATCAATAAGCGGAGGAAAAGAAACCAAA

>*I. indigotica*（17）

GGGGGCCCTTGGTGGATGAGACGTAGCGAATGCGATACTTGGTGTGAATTGCAGAATCCCGTGAACCATCGAGTCTTTGAACGCAAGTTGCGCCCTAAGCCTTCTGGCCGAGGGCACGTCTGCCTGGGTGTCACAAATCGTCGTCCCCCCATCCTCTCGAGGATAATGGACGGAAGCTGGTCTCCCGTGTGTTACCGCACGCGGTTGGCCAAAATCCGAGCTAAGGACGCAAGGAGCGTCTCGACATGCGGTGGTGAATTAAAACCTCGTCATACCGTTGGCCGCTCCTGTCCTGATGCTCTCGATGACCCAAAGTCCTCAACGCGACCCCAGGTCAGGCGGGATCACCCGCTGAGTTTAAGCATATCAATAAGCGGAGGAAAAGAAACTAAA

>*I. indigotica*（18）

GCCCCGGGGACCGATGAGACGTAGCGAATGCGATACTTGGTGTGAATTGCAGAATCCCGTGAACCATCGAGTCTTTGAACGCAAGTTGCGCCCTAAGCCTTCTGGCCGAGGGCACGTCTGCCTGGGTGTCACAAATCGTCGTCCCCCCATCCTCTCGAGGATAATGGACGGAAGCTGGTCTCCCGTGTGTTACCGCACGCGGTTGGCCAAAATCCGAGCTAAGGACGCAAGGAGCGTCTCGACATGCGGTGGTGAATTAAAACCTCGTCATACCGTTGGCCGCTCCTGTCCTGATGCTCTCGATGACCCAAAGTCCTCAACGCGACCCCAGGTCAGGCGGGATCACCCGCTGAGTTTAAGCATATCAATAAGCGGAGGAAAAGAAACTAA

>*I. indigotica*（19）

AGGGCCGCTCGGTGGATGAGACGTAGCGAATGCGATACTTGGTGTGAATTGCAGAATCCCGTGAACCATCGAGTCTTTGAACGCAAGTTGCGCCCTAAGCCTTCTGGCCGAGGGCACGTCTGCCTGGGTGTCACAAATCGTCGTCCCCCCATCCTCTCGAGGATAATGGACGGAAGCTGGTCTCCCGTGTGTTACCGCACGCGGTTGGCCAAAATCCGAGCTAAGGACGCAAGGAGCGTCTCGACATGCGGTGGTGAATTAAAACCTCGTCATACCGTTGGCCGCTCCTGTCCTGATGCTCTCGATGACCCAAAGTCCTCAACGCGACCCCAGGTCAGGCGGGATCACCCGCTGAGTTTAAGCATATCAATAAGCGGAGGAAAAGAAACTAA

>*I. indigotica*（20）

GCTTCTAGGTTCGACTGAGAGAGACGTAGCGAATGCGATCTTGGTGTGATTGCAGAATCCCGTGAACCATCGAGTCTTTGAACGCAAGTTGCGCCCTAAGCCTTCTGGCCGAGGGCACGTCTGCCTGGGTGTCACAAATCGTCGTCCCCCCATCCTCTCGAGGATAATGGACGGAAGCTGGTCTCCCGTGTGTTACCGCACGCGGTTGGCCAAAATCCGAGCTAAGGACGCAAGGAGCGTCTCGACATGCGGTGGTGAATTAAAACCTCGTCATACCGTTGGCCGCTCCTGTCCTGATGCTCTCGATGACCCAAAGTCCTCAACGCGACCCCAGGTCAGGCGGGATCACCCGCTGAGTTTAAGCATATCAATAAGCGGAGGAAAAGAAACTAAA

>*I. indigotica*（21）

CCCCGGCGGATTGATGAGGACGTAGCGAATGCGATACTTGGTGTGAATTGCAGAATCCCGTGAACCATCGAGTCTTTGAACGCAAGTTGCGCCCTAAGCCTTCTGGCCGAGGGCACGTCTGCCTGGGTGTCACAAATCGTCGTCCCCCCATCCTCTCGAGGATAATGGACGGAAGCTGGTCTCCCGTGTGTTACCGCACGCGGTTGGCCAAAATCCGAGCTAAGGACGCAAGGAGCGTCTCGACATGCGGTGGTGAATTAAAACCTCGTCATACCGTTGGCCGCTCCTGTCCTGATGCTCTCGATGACCCAAAGTCCTCAACGCGACCCCAGGTCAGGCGGGATCACCCGCTGAGTTTAAGCATATCAATAAGCGGAGGAAAAGAAACTAAA

>*I. indigotica*（22）

GCGGGTGATGATGAGACGTAGCGATGCGATACTTGGTGTGATTGCAGAATCCCGTGAACCATCGAGTCTTTGAACGCAAGTTGCGCCCTAAGCCTTCTGGCCGAGGGCACGTCTGCCTGGGTGTCACAAATCGTCGTCCCCCCATCCTCTCGAGGATAATGGACGGAAGCTGGTCTCCCGTGTGTTACCGCACGCGGTTGGCCAAAATCCGAGCTAAGGACGCAAGGAGCGTCTCGACATGCGGTGGTGAATTAAAACCTCGTCATACCGTTGGCCGCTCCTGTCCTGATGCTCTCGATGACCCAAAGTCCTCAACGCGACCCCAGGTCAGGCGGGATCACCCGCTGAGTTTAAGCATATCAATAAGCGGAGGAAAAGAAACCAAATT

>*I. indigotica*（23）

GCCACGATGAGACGTAGCGATGCGATACTTGGTGTGAATTGCAGAATCCCGTGAACCATCGAGTCTTTGAACGCAAGTTGCGCCCTAAGCCTTCTGGCCGAGGGCACGTCTGCCTGGGTGTCACAAATCGTCGTCCCCCCATCCTCTCGAGGATAATGGACGGAAGCTGGTCTCCCGTGTGTTACCGCACGCGGTTGGCCAAAATCCGAGCTAAGGACGCAAGGAGCGTCTCGACATGCGGTGGTGAATTAAAACCTCGTCATACCGTTGGCCGCTCCTGTCCTGATGCTCTCGATGACCCAAAGTCCTCAACGCGACCCCAGGTCAGGCGGGATCACCCGCTGAGTTTAAGCATATCAATAAGCGGAGAAAAAGAAACTAAAAC

>*I. indigotica*（24）

TTAACGATGAGACGTAGCGATGCGATACTTGGTGTGAATTGCAGAATCCCGTGAACCATCGAGTCTTTGAACGCAAGTTGCGCCCTAAGCCTTCTGGCCGAGGGCACGTCTGCCTGGGTGTCACAAATCGTCGTCCCCCCATCCTCTCGAGGATAATGGACGGAAGCTGGTCTCCCGTGTGTTACCGCACGCGGTTGGCCAAAATCCGAGCTAAGGACGCAAGGAGCGTCTCGACATGCGGTGGTGAATTAAAACCTCGTCATACCGTTGGCCGCTCCTGTCCTGATGCTCTCGATGACCCAAAGTCCTCAACGCGACCCCAGGTCAGGCGGGATCACCCGCTGAGTTTAAGCATATCAATAAGCGGAGAAAAAAGAAACTAA

>*I. indigotica*（25）

CCACGATGAGACGTAGCGATGCGATACTTGGTGTGAATTGCAGAATCCCGTGAACCATCGAGTCTTTGAACGCAAGTTGCGCCCTAAGCCTTCTGGCCGAGGGCACGTCTGCCTGGGTGTCACAAATCGTCGTCCCCCCATCCTCTCGAGGATAATGGACGGAAGCTGGTCTCCCGTGTGTTACCGCACGCGGTTGGCCAAAATCCGAGCTAAGGACGCAAGGAGCGTCTCGACATGCGGTGGTGAATTAAAACCTCGTCATACCGTTGGCCGCTCCTGTCCTGATGCTCTCGATGACCCAAAGTCCTCAACGCGACCCCAGGTCAGGCGGGATCACCCGCTGAGTTTAAGCATATCAATAAGCGGAGAAA

>*I. indigotica*（26）

CGAAAGCACGATGAGACGTAGCGATGCGATACTTGGTGTGAATTGCAGAATCCCGTGAACCATCGAGTCTTTGAACGCAAGTTGCGCCCTAAGCCTTCTGGCCGAGGGCACGTCTGCCTGGGTGTCACAAATCGTCGTCCCCCCATCCTCTCGAGGATAATGGACGGAAGCTGGTCTCCCGTGTGTTACCGCACGCGGTTGGCCAAAATCCGAGCTAAGGACGCAAGGAGCGTCTCGACATGCGGTGGTGAATTAAAACCTCGTCATACCGTTGGCCGCTCCTGTCCTGATGCTCTCGATGACCCAAAGTCCTCAACGCGACCCCAGGTCAGGCGGGATCACCCGCTGAGTTTAAGCATATCAATAAGCGGAGGAAAAGAAACCAAAAC

>*I. indigotica*（27）

TGGTGACGATGAGACGTAGCGATGCGATACTTGGTGTGAATTGCAGAATCCCGTGAACCATCGAGTCTTTGAACGCAAGTTGCGCCCTAAGCCTTCTGGCCGAGGGCACGTCTGCCTGGGTGTCACAAATCGTCGTCCCCCCATCCTCTCGAGGATAATGGACGGAAGCTGGTCTCCCGTGTGTTACCGCACGCGGTTGGCCAAAATCCGAGCTAAGGACGCAAGGAGCGTCTCGACATGCGGTGGTGAATTAAAACCTCGTCATACCGTTGGCCGCTCCTGTCCTGATGCTCTCGATGACCCAAAGTCCTCAACGCGACCCCAGGTCAGGCGGGATCACCCGCTGAGTTTAAGCATATCAATAAGCGGAGAAAAAGAAACTAAAAGA

>*I. tinctoria*（28）

AGGTTGAATCGATGAGACGTAGCGATGCGATACTTGGTGTGAATTGCAGAATCCCGTGAACCATCGAGTCTTTGAACGCAAGTTGCGCCCTAAGCCTTCTGGCCGAGGGCACGTCTGCCTGGGTGTCACAAATCGTCGTCCCCCCATCCTCTCGAGGATAATGGACGGAAGCTGGTCTCCCGTGTGTTACCGCACGCGGTTGGCCAAAATCCGAGCTAAGGACGCAAGGAGCGTCCCGACATGCGGTGGTGAATTAAAACCTCGTCATACCGTCGGCCGCTCCTGTCCTGATGCTCTCGATGACCCAATGTCCTCAACGCGACCCCAGGTCAGGCGGGATCACCCGCTGAGTTTAAGCATATCAATAAGCGGAGGAAAAGAAACCAAAC

**Sequence of mini-barcode(1~28)**

>*I. indigotica* (Wanrong)

TCTTGTATTTCTTCGTTAAGAGGAAATACAAGATCTTTAAGCGAAATCAATAGATTCATTAATTTAAGTGCAAAATTTTGCACTTTATATCTTTAGGAATTATTATTTCTCTGAATTCTTTTATTCTTTAACTTGGAATCAAAATTATAAAAGCATCTATCCACAACAAGTG-------------AATATTAGATAATAAGAATAAATAATAATATATGATATTATTTTTAAATGATCAAAAAATCTTATGTTTGTACAATATAAAAAGATGCATGAAAGTAGATATTTTGAGAATTATTATTATTATTTTTTTCATTTCTCTTGAGCAACTTAGGCAAATCTTATTTTATTTCAAATTTCTAA

>*I. indigotica* (Altay)

TCTTGTATTTCTTCGTTAAGAGGAAATACAAGATCTTTAAGCGAAATCAATAGATTCATTAATTTAAGTGCAAAATTTTGCACTTTATATCTTTAGGAATTATTATTTCTCTGAATTCTTTTATTCTTTAACTTGGAATCAAAATTATAAAAGCATCTATCCACAACAAGTG-------------AATATTAGATAATAAGAATAAATAATAATATATGATATTATTTTTAAATGATCAAAAAATCTTATGTTTGTACAATATAAAAAGATGCATGAAAGTAGATATTTTGAGAATTATTATTATTATTTTTTTCATTTCTCTTGAGCAACTTAGGCAAATCTTATTTTATTTCAAATTTCTAA

>*I. indigotica* (Ruicheng)

TCTTGTATTTCTTCGTTACGAGGAAATACAAGATCTTTAAGCGAAATCAATAGATTCATTAATTTAAGTGCAAAATTTTGCACTTTATATCTTTAGGAATTATTATTTCTCTGAATTCTTTTATTCTTTAACTTGGAATCAAAATTATAAAAGCATCTATCCACAACAAGTG-------------AATATTAGATAATAAGAATAAATAATAATATATGATATTATTTTTAAATGATCAAAAAATCTTATGTTTGTACAATATAAAAAGATGCATGAAAGTAGATATTTTGAGAATTATTATTATTATTTTTTTCATTTCTCTTGAGCAACTTAGGCAAATCTTATTTTATTTCAAATTTCTAA

>*I. indigotica* (Linfen)

TCTTGTATTTCTTCGTTAAGAGGAAATACAAGATCTTTAAGCGAAATCAATAGATTCATTAATTTAAGTGCAAAATTTTGCACTTTATATCTTTAGGAATTATTATTTCTCTGAATTCTTTTATTCTTTAACTTGGAATCAAAATTATAAAAGCATCTATCCACAACAAGTG-------------AATATTAGATAATAAGAATAAATAATAATATATGATATTATTTTTAAATGATCAAAAAATCTTATGTTTGTACAATATAAAAAGATGCATGAAAGTAGATATTTTGAGAATTATTATTATTATTTTTTTCATTTCTCTTGAGCAACTTAGGCAAATCTTATTTTATTTCAAATTTCTAA

>*I. indigotica* (Ruanqiao)

TCTTGTATTTCTTCTTAACGAAGAAATACAAGATCTTTAAGCGAAATCAATAGATTCATTAATTTAAGTGCAAAATTTTGCACTTTATATCTTTAGGAATTATTATTTCTCTGAATTCTTTTATTCTTTAACTTGGAATCAAAATTATAAAAGCATCTATCCACAACAAGTG-------------AATATTAGATAATAAGAATAAATAATAATATATGATATTATTTTTAAATGATCAAAAAATCTTATGTTTGTACAATATAAAAAGATGCATGAAAGTAGATATTTTGAGAATTATTATTATTATTTTTTTCATTTCTCTTGAGCAACTTAGGCAAATCTTATTTTATTTCAAATTTCTAA

>*I. indigotica* (Bozhou)

TCTTGTATTTCTTCGTTAAGAGGAAATACAAGATCTTTAAGCGAAATCAATAGATTCATTAATTTAAGTGCAAAATTTTGCACTTTATATCTTTAGGAATTATTATTTCTCTGAATTCTTTTATTCTTTAACTTGGAATCAAAATTATAAAAGCATCTATCCACAACAAGTG-------------AATATTAGATAATAAGAATAAATAATAATATATGATATTATTTTTAAATGATCAAAAAATCTTATGTTTGTACAATATAAAAAGATGCATGAAAGTAGATATTTTGAGAATTATTATTATTATTTTTTTCATTTCTCTTGAGCAACTTAGGCAAATCTTATTTTATTTCAAATTTCTAA

>*I. indigotica* (Baoding)

TCTTGTATTTCTTCGTTAAGAGGAAATACAAGATCTTTAAGCGAAATCAATAGATTCATTAATTTAAGTGCAAAATTTTGCACTTTATATCTTTAGGAATTATTATTTCTCTGAATTCTTTTATTCTTTAACTTGGAATCAAAATTATAAAAGCATCTATCCACAACAAGTG-------------AATATTAGATAATAAGAATAAATAATAATATATGATATTATTTTTAAATGATCAAAAAATCTTATGTTTGTACAATATAAAAAGATGCATGAAAGTAGATATTTTGAGAATTATTATTATTATTTTTTTCATTTCTCTTGAGCAACTTAGGCAAATCTTATTTTATTTCAAATTTCTAA

>*I. indigotica* (Guannan)

TCTTGTATTTCTTCTTAACGAAGAAATACAAGATCTTTAAGCGAAATCAATAGATTCATTAATTTAAGTGCAAAATTTTGCACTTTATATCTTTAGGAATTATTATTTCTCTGAATTCTTTTATTCTTTAACTTGGAATCAAAATTATAAAAGCATCTATCCACAACAAGTG-------------AATATTAGATAATAAGAATAAATAATAATATATGATATTATTTTTAAATGATCAAAAAATCTTATGTTTGTACAATATAAAAAGATGCATGAAAGTAGATATTTTGAGAATTATTATTATTATTTTTTTCATTTCTCTTGAGCAACTTAGGCAAATCTTATTTTATTTCAAATTTCTAA

>*I. indigotica* (Luoyang)

TCTTGTATTTCTTCGTTAAGAGGAAATACAAGATCTTTAAGCGAAATCAATAGATTCATTAATTTAAGTGCAAAATTTTGCACTTTATATCTTTAGGAATTATTATTTCTCTGAATTCTTTTATTCTTTAACTTGGAATCAAAATTATAAAAGCATCTATCCACAACAAGTG-------------AATATTAGATAATAAGAATAAATAATAATATATGATATTATTTTTAAATGATCAAAAAATCTTATGTTTGTACAATATAAAAAGATGCATGAAAGTAGATATTTTGAGAATTATTATTATTATTTTTTTCATTTCTCTTGAGCAACTTAGGCAAATCTTATTTTATTTCAAATTTCTAA

>*I. indigotica* (Jiaozuo)

TCTTGTATTTCCTCTTAACGAGGAAATACAAGATCTTTAAGCGAAATCAATAGATTCATTAATTTAAGTGCAAAATTTTGCACTTTATATCTTTAGGAATTATTATTTCTCTGAATTCTTTTATTCTTTAACTTGGAATCAAAATTATAAAAGCATCTATCCACAACAAGTG-------------AATATTAGATAATAAGAATAAATAATAATATATGATATTATTTTTAAATGATCAAAAAATCTTATGTTTGTACAATATAAAAAGATGCATGAAAGTAGATATTTTGAGAATTATTATTATTATTTTTTTCATTTCTCTTGAGCAACTTAGGCAAATCTTATTTTATTTCAAATTTCTAA

>*I. indigotica* (Qixian)

TCTTGTATTTCTTCGTTAAGAGGAAATACAAGATCTTTAAGCGAAATCAATAGATTCATTAATTTAAGTGCAAAATTTTGCACTTTATATCTTTAGGAATTATTATTTCTCTGAATTCTTTTATTCTTTAACTTGGAATCAAAATTATAAAAGCATCTATCCACAACAAGTG-------------AATATTAGATAATAAGAATAAATAATAATATATGATATTATTTTTAAATGATCAAAAAATCTTATGTTTGTACAATATAAAAAGATGCATGAAAGTAGATATTTTGAGAATTATTATTATTTTTTTTTTCATTTCTCTTGAGCAACTTAGGCAAATCTTATTTTATTTCAAATTTCTAA

>*I. indigotica* (ruzhou)

TCTTGTATTTCTTCGTTAAGAGGAAATACAAGATCTTTAAGCGAAATCAATAGATTCATTAATTTAAGTGCAAAATTTTGCACTTTATATCTTTAGGAATTATTATTTCTCTGAATTCTTTTATTCTTTAACTTGGAATCAAAATTATAAAAGCATCTATCCACAACAAGTG-------------AATATTAGATAATAAGAATAAATAATAATATATGATATTATTTTTAAATGATCAAAAAATCTTATGTTTGTACAATATAAAAAGATGCATGAAAGTAGATATTTTGAGAATTATTATTATTATTTTTTTCATTTCTCTTGAGCAACTTAGGCAAATCTTATTTTATTTCAAATTTCTAA

>*I. indigotica* (Lingbao)

TCTTGTATTTCTTCGTTAAGAGGAAATACAAGATCTTTGTTTATTATGCAATCAAGGTATAATTTAAGTGCAAAATTTTGCACTTTATATCTTTAGGAATTATTATTTCTCTGAATTCTTTTATTCTTTAACTTGGAATCAAAATTATAAAAGCATCTATCCACAACAAGTG-------------AATATTAGATAATAAGAATAAATAATAATATATGATATTATTTTTAAATGATCAAAAAATCTTATGTTTGTACAATATAAAAAGATGCATGAAAGTAGATATTTTGAGAATTATTATTATTATTTTTTTCATTTCTCTTGAGCAACTTAGGCAAATCTTATTTTATTTCAAATTTCTAA

>*I. indigotica* (Wenan)

TCTTGTATTTCTTCTTTAAGAAGAAATACAAGATCTTTAAGCGAAATCAATAGATTCATTAATTTAAGTGCAAAATTTTGCACTTTATATCTTTAGGAATTATTATTTCTCTGAATTCTTTTATTCTTTAACTTGGAATCAAAATTATAAAAGCATCTATCCACAACAAGTG-------------AATATTAGATAATAAGAATAAATAATAATATATGATATTATTTTTAAATGATCAAAAAATCTTATGTTTGTACAATATAAAAAGATGCATGAAAGTAGATATTTTGAGAATTATTATTATTATTTTTTTCATTTCTCTTGAGCAACTTAGGCAAATCTTATTTTATTTCAAATTTCTAA

>*I. indigotica* (Shangluo)

TCTTGTATTTCTTCGTTAAGAGGAAATACAAGATCTTTAAGCGAAATCAATAGATTCATTAATTTAAGTGCAAAATTTTGCACTTTATATCTTTAGGAATTATTATTTCTCTGAATTCTTTTATTCTTTAACTTGGAATCAAAATTATAAAAGCATCTATCCACAACAAGTG-------------AATATTAGATAATAAGAATAAATAATAATATATGATATTATTTTTAAATGATCAAAAAATCTTATGTTTGTACAATATAAAAAGATGCATGAAAGTAGATATTTTGAGAATTATTATTATTATTTTTTTCATTTCTCTTGAGCAACTTAGGCAAATCTTATTTTATTTCAAATTTCTAA

>*I. indigotica* (Longde)

TCTTGTATTTCTTCGTTAAGAGGAAATACAAGATCTTTAAGCGAAATCAATAGATTCATTAATTTAAGTGCAAAATTTTGCACTTTATATCTTTAGGAATTATTATTTCTCTGAATTCTTTTATTCTTTAACTTGGAATCAAAATTATAAAAGCATCTATCCACAACAAGTG-------------AATATTAGATAATAAGAATAAATAATAATATATGATATTATTTTTAAATGATCAAAAAATCTTATGTTTGTACAATATAAAAAGATGCATGAAAGTAGATATTTTGAGAATTATTATTATTATTTTTTTCATTTCTCTTGAGCAACTTAGGCAAATCTTATTTTATTTCAAATTTCTAA

>*I. indigotica* (Zhangye)

TCTTGTATTTCTTCGTTAAGAGGAAATACAAGATCTTTAAGCGAAATCAATAGATTCATTAATTTAAGTGCAAAATTTTGCACTTTATATCTTTAGGAATTATTATTTCTCTGAATTCTTTTATTCTTTAACTTGGAATCAAAATTATAAAAGCATCTATCCACAACAAGTG-------------AATATTAGATAATAAGAATAAATAATAATATATGATATTATTTTTAAATGATCAAAAAATCTTATGTTTGTACAATATAAAAAGATGCATGAAAGTAGATATTTTGAGAATTATTATTATTTTTTTTTTCATTTCTCTTGAGCAACTTAGGCAAATCTTATTTTATTTCAAATTTCTAA

>*I. indigotica* (Longxi)

TCTTGTATTTCTTCGTTAAGAGGAAATACAAGATCTTTAAGCGAAATCAATAGATTCATTAATTTAAGTGCAAAATTTTGCACTTTATATCTTTAGGAATTATTATTTCTCTGAATTCTTTTATTCTTTAACTTGGAATCAAAATTATAAAAGCATCTATCCACAACAAGTG-------------AATATTAGATAATAAGAATAAATAATAATATATGATATTATTTTTAAATGATCAAAAAATCTTATGTTTGTACAATATAAAAAGATGCATGAAAGTAGATATTTTGAGAATTATTATTATTATTTTTTTCATTTCTCTTGAGCAACTTAGGCAAATCTTATTTTATTTCAAATTTCTAA

>*I. indigotica* (Zhangshu)

TCTTGTATTTCTTCGTTAAGAGGAAATACAAGATCTTTAAGCGAAATCAATAGATTCATTAATTTAAGTGCAAAATTTTGCACTTTATATCTTTAGGAATTATTATTTCTCTGAATTCTTTTATTCTTTAACTTGGAATCAAAATTATAAAAGCATCTATCCACAACAAGTG-------------AATATTAGATAATAAGAATAAATAATAATATATGATATTATTTTTAAATGATCAAAAAATCTTATGTTTGTACAATATAAAAAGATGCATGAAAGTAGATATTTTGAGAATTATTATTATTATTTTTTTCATTTCTCTTGAGCAACTTAGGCAAATCTTATTTTATTTCAAATTTCTAA

>*I. indigotica* (Midu)

TCTTGTATTTCTTCGTTAAGAGGAAATACAAGATCTTTAAGCGAAATCAATAGATTCATTAATTTAAGTGCAAAATTTTGCACTTTATATCTTTAGGAATTATTATTTCTCTGAATTCTTTTATTCTTTAACTTGGAATCAAAATTATAAAAGCATCTATCCACAACAAGTG-------------AATATTAGATAATAAGAATAAATAATAATATATGATATTATTTTTAAATGATCAAAAAATCTTATGTTTGTACAATATAAAAAGATGCATGAAAGTAGATATTTTGAGAATTATTATTATTATTTTTTTCATTTCTCTTGAGCAACTTAGGCAAATCTTATTTTATTTCAAATTTCTAA

>*I. indigotica* (Hotan)

TCTTGTATTTCTTCGTTAAGAGGAAATACAAGATCTTTAAGCGAAATCAATAGATTCATTAATTTAAGTGCAAAATTTTGCACTTTATATCTTTAGGAATTATTATTTCTCTGAATTCTTTTATTCTTTAACTTGGAATCAAAATTATAAAAGCATCTATCCACAACAAGTG-------------AATATTAGATAATAAGAATAAATAATAATATATGATATTATTTTTAAATGATCAAAAAATCTTATGTTTGTACAATATAAAAAGATGCATGAAAGTAGATATTTTGAGAATTATTATTATTATTTTTTTCATTTCTCTTGAGCAACTTAGGCAAATCTTATTTTATTTCAAATTTCTAA

>*I. indigotica* (Fuyang)

TCTTGTATTTCTTCGTTAAGAGGAAATACAAGATCTTTAAGCGAAATCAATAGATTCATTAATTTAAGTGCAAAATTTTGCACTTTATATCTTTAGGAATTATTATTTCTCTGAATTCTTTTATTCTTTAACTTGGAATCAAAATTATAAAAGCATCTATCCACAACAAGTG-------------AATATTAGATAATAAGAATAAATAATAATATATGATATTATTTTTAAATGATCAAAAAATCTTATGTTTGTACAATATAAAAAGATGCATGAAAGTAGATATTTTGAGAATTATTATTATTATTTTTTTCATTTCTCTTGAGCAACTTAGGCAAATCTTATTTTATTTCAAATTTCTAA

>*I. indigotica* (Bozhou)

TCTTGTATTTCTTCGTTAAGAGGAAATACAAGATCTTTAAGCGAAATCAATAGATTCATTAATTTAAGTGCAAAATTTTGCACTTTATATCTTTAGGAATTATTATTTCTCTGAATTCTTTTATTCTTTAACTTGGAATCAAAATTATAAAAGCATCTATCCACAACAAGTG-------------AATATTAGATAATAAGAATAAATAATAATATATGATATTATTTTTAAATGATCAAAAAATCTTATGTTTGTACAATATAAAAAGATGCATGAAAGTAGATATTTTGAGAATTATTATTATTATTTTTTTCATTTCTCTTGAGCAACTTAGGCAAATCTTATTTTATTTCAAATTTCTAA

>*I. indigotica* (Daqing)

TCTTGTATTTCTTCGTTAAGAGGAAATACAAGATCTTTAAGCGAAATCAATAGATTCATTAATTTAAGTGCAAAATTTTGCACTTTATATCTTTAGGAATTATTATTTCTCTGAATTCTTTTATTCTTTAACTTGGAATCAAAATTATAAAAGCATCTATCCACAACAAGTG-------------AATATTAGATAATAAGAATAAATAATAATATATGATATTATTTTTAAATGATCAAAAAATCTTATGTTTGTACAATATAAAAAGATGCATGAAAGTAGATATTTTGAGAATTATTATTATTATTTTTTTCATTTCTCTTGAGCAACTTAGGCAAATCTTATTTTATTTCAAATTTCTAA

>*I. indigotica* (Xingtai)

TCTTGTATTTCTTCGTTAAGAGGAAATACAAGATCTTTAAGCGAAATCAATAGATTCATTAATTTAAGTGCAAAATTTTGCACTTTATATCTTTAGGAATTATTATTTCTCTGAATTCTTTTATTCTTTAACTTGGAATCAAAATTATAAAAGCATCTATCCACAACAAGTG-------------AATATTAGATAATAAGAATAAATAATAATATATGATATTATTTTTAAATGATCAAAAAATCTTATGTTTGTACAATATAAAAAGATGCATGAAAGTAGATATTTTGAGAATTATTATTATTATTTTTTTCATTTCTCTTGAGCAACTTAGGCAAATCTTATTTTATTTCAAATTTCTAA

>*I. indigotica* (Shanghai)

TCTTGTATTTCTTCGTTAAGAGGAAATACAAGATCTTTAAGCGAAATCAATAGATTCATTAATTTAAGTGCAAAATTTTGCACTTTATATCTTTAGGAATTATTATTTCTCTGAATTCTTTTATTCTTTAACTTGGAATCAAAATTATAAAAGCATCTATCCACAACAAGTG-------------AATATTAGATAATAAGAATAAATAATAATATATGATATTATTTTTAAATGATCAAAAAATCTTATGTTTGTACAATATAAAAAGATGCATGAAAGTAGATATTTTGAGAATTATTATTATTATTTTTTTCATTTCTCTTGAGCAACTTAGGCAAATCTTATTTTATTTCAAATTTCTAA

>*I. indigotica*(Qianxian)-tetraploid

TCTTGTATTTCTTCGTTAAGAGGAAATACAAGATCTTTAAGCGAAATCAATAGATTCATTAATTTAAGTGCAAAATTTTGCACTTTATATCTTTAGGAATTATTATTTCTCTGAATTCTTTTATTCTTTAACTTGGAATCAAAATTATAAAAGCATCTATCCACAACAAGTG-------------AATATTAGATAATAAGAATAAATAATAATATATGATATTATTTTTAAATGATCAAAAAATCTTATGTTTGTACAATATAAAAAGATGCATGAAAGTAGATATTTTGAGAATTATTATTATTTTTTTTTTCATTTCTCTTGAGCAACTTAGGCAAATCTTATTTTATTTCAAATTTCTAA

>*I. tinctoria* explant(1)

TCTTGTATTTTTTCGTTAAGAGGAAATACAAGATCTTTAAGCGAAATCAATAGATTCATTAATTTAAGTGCAAAATTTTGCACTTTATATCTTTAGGAATTATTATTTCTCTTAATTCTTTTATTCTTTA----GGAATCAAAATTATAAAAGCATCTATCCACAACAAGTGAATATTAGATAATAATATTAGATAATAATAATAAATAATAATATATGATATTATTTTTAAATGATCAAAAAATCTTATGTTTGTACAATATAAAAAGATGCATGAAAGTATATATTTTGATAATTATTATTATTTTTTTTTTCATTTCTCTTGAGCAACTTAGGCAAATCTTATTTTATTTCAAATTTCTAA

>*I. tinctoria* (2)

TCTTGTATTTTTTCGTTAAGAGGAAATACAAGATCTTTAAGCGAAATCAATAGATTCATTAATTTAAGTGCAAAATTTTGCACTTTATATCTTTAGGAATTATTATTTCTCTTAATTCTTTTATTCTTTA----GGAATCAAAATTATAAAAGCATCTATCCACAACAAGTGAATATTAGATAATAATATTAGATAATAATAATAAATAATAATATATGATATTATTTTTAAATGATCAAAAAATCTTATGTTTGTACAATATAAAAAGATGCATGAAAGTATATATTTTGATAATTATTATTATTTTTTTTTTCATTTCTCTTGAGCAACTTAGGCAAATCTTATTTTATTTCAAATTTCTAA

>*I. tinctoria* (3)

TCTTGTATTTCTTCGTTAAGAGGAAATACAAGATCTTTAAGCGAAATCAATAGATTCATTAATTTAAGTGCAAAATTTTGCACTTTATATCTTTAGGAATTATTATTTCTCTTAATTCTTTTATTCTTTA----GGAATCAAAATTATAAAAGCATCTATCCACAACAAGTGAATATTAGATAATAATATTAGATAATAATAATAAATAATAATATATGATATTATTTTTAAATGATCAAAAAATCTTATGTTTGTACAATATAAAAAGATGCATGAAAGTATATATTTTGATAATTATTATTATTTTTTTTTTCATTTCTCTTGAGCAACTTAGGCAAATCTTATTTTATTTCAAATTTCTAA
